# Climate-change-enhanced habitat diversification for the Middle Jurassic Yanliao Biota in East Asia

**DOI:** 10.1093/nsr/nwaf194

**Published:** 2025-05-16

**Authors:** Wenxing Hao, Jianghai Yang, Haibing Wang, Ross N Mitchell, Chunxia Zhang, Ruoyuan Qiu, Jiaqi Guo, Wang Zhang, Xiujuan Bao, Chenglong Deng, Xiaolin Wang, Yongyun Hu, Jin-Hui Yang, Guang Zhu, Zhonghe Zhou, Rixiang Zhu

**Affiliations:** State Key Laboratory of Lithospheric and Environmental Coevolution, Institute of Geology and Geophysics, Chinese Academy of Sciences, Beijing 100029, China; National Key Laboratory of Deep Oil and Gas, School of Geosciences, China University of Petroleum (East China), Qingdao 266580, China; State Key Laboratory of Geomicrobiology and Environmental Changes, School of Earth Sciences, China University of Geosciences, Wuhan 430074, China; Key Laboratory of Vertebrate Evolution and Human Origins of Chinese Academy of Sciences, Institute of Vertebrate Paleontology and Paleoanthropology, Chinese Academy of Sciences, Beijing 100044, China; State Key Laboratory of Lithospheric and Environmental Coevolution, Institute of Geology and Geophysics, Chinese Academy of Sciences, Beijing 100029, China; Key Laboratory of Cenozoic Geology and Environment, Institute of Geology and Geophysics, Chinese Academy of Sciences, Beijing 100029, China; State Key Laboratory of Lithospheric and Environmental Coevolution, Institute of Geology and Geophysics, Chinese Academy of Sciences, Beijing 100029, China; Laboratory for Climate and Ocean–Atmosphere Studies, Department of Atmospheric and Oceanic Sciences, School of Physics, Peking University, Beijing 100871, China; State Key Laboratory of Lithospheric and Environmental Coevolution, Institute of Geology and Geophysics, Chinese Academy of Sciences, Beijing 100029, China; State Key Laboratory of Lithospheric and Environmental Coevolution, Institute of Geology and Geophysics, Chinese Academy of Sciences, Beijing 100029, China; State Key Laboratory of Lithospheric and Environmental Coevolution, Institute of Geology and Geophysics, Chinese Academy of Sciences, Beijing 100029, China; Key Laboratory of Vertebrate Evolution and Human Origins of Chinese Academy of Sciences, Institute of Vertebrate Paleontology and Paleoanthropology, Chinese Academy of Sciences, Beijing 100044, China; Laboratory for Climate and Ocean–Atmosphere Studies, Department of Atmospheric and Oceanic Sciences, School of Physics, Peking University, Beijing 100871, China; State Key Laboratory of Lithospheric and Environmental Coevolution, Institute of Geology and Geophysics, Chinese Academy of Sciences, Beijing 100029, China; School of Resource and Environmental Engineering, Hefei University of Technology, Hefei 230009, China; Key Laboratory of Vertebrate Evolution and Human Origins of Chinese Academy of Sciences, Institute of Vertebrate Paleontology and Paleoanthropology, Chinese Academy of Sciences, Beijing 100044, China; State Key Laboratory of Lithospheric and Environmental Coevolution, Institute of Geology and Geophysics, Chinese Academy of Sciences, Beijing 100029, China

**Keywords:** Yanliao Biota, Middle Jurassic, North China Craton

## Abstract

The Jurassic Period was characterized by the dominance of dinosaurs and the rise of early mammals, with the Yanliao Biota (∼167–157 Ma) in East Asia notable for its exceptional fossil preservation and diverse life forms. However, the drivers of the flourishing of the Yanliao Biota remain unclear. Here, we reconstruct the palaeoclimate and habitat characteristics of the Yanliao Biota by using sedimentary facies analysis, organic carbon-isotope data, palynological records, source weathering trends and climate simulations from the Community Earth System Model CESM 1.2.2. The studied sedimentary successions are well constrained to the Middle Jurassic based on carbon-isotope stratigraphic correlation and published tuff zircon U–Pb ages. Our findings reveal a regional climate shift in the late Bathonian, transitioning from wet to sub-humid conditions, as evidenced by an increase in gymnosperm pollen, a marked decline in coal seams and reduced weathering intensity. Sedimentological evidence further supports a synchronous facies change from ever-wet fluvial-delta systems to seasonally active alluvial plains. This climate shift aligns with simulation results and coincides temporally with the initial flourishing of the Yanliao Biota. We propose that this shift, associated with lithospheric extension in the Yanliao region, increased landscape heterogeneity and habitat diversity, fostering biological evolution through ecological isolation and allopatric speciation, ultimately driving the diversification of the Yanliao Biota.

## Teaser

A late Bathonian climate shift from wet to sub-humid conditions in the Yanliao region increased habitat diversity, driving Yanliao Biota diversification through ecological isolation and allopatric speciation.

## INTRODUCTION

The evolution of terrestrial life since the Ordovician represents one of the most transformative developments in Earth's history, fundamentally altering the planet's ecosystems and biodiversity [1]. The Jurassic Period was marked by significant evolutionary events and the flourishing of diverse terrestrial biota [2]. During this period, mammalian diversity was in its early stage of evolution and palaeoflora ecosystems differed substantially from those of the present day [3]. In East Asia, one of the most important biological events was the flourishing of the Yanliao Biota (∼167–157 Ma) during the Middle to Late Jurassic in the North China Craton (NCC) [4,5]. This Biota records the earliest diversification of mammals, marked by specialized ecological adaptations such as swimming, arboreal lifestyles, gliding and digging behaviours [6,7]. It also preserves exceptional dinosaurs that illustrate the evolutionary transition from dinosaurs to birds, alongside insect fossils that contribute to understanding insect evolution [8]. This ecological richness, particularly among mammals, establishes the Yanliao Biota as an iconic milestone in the Mesozoic biotic record [4].

**Figure 1.**
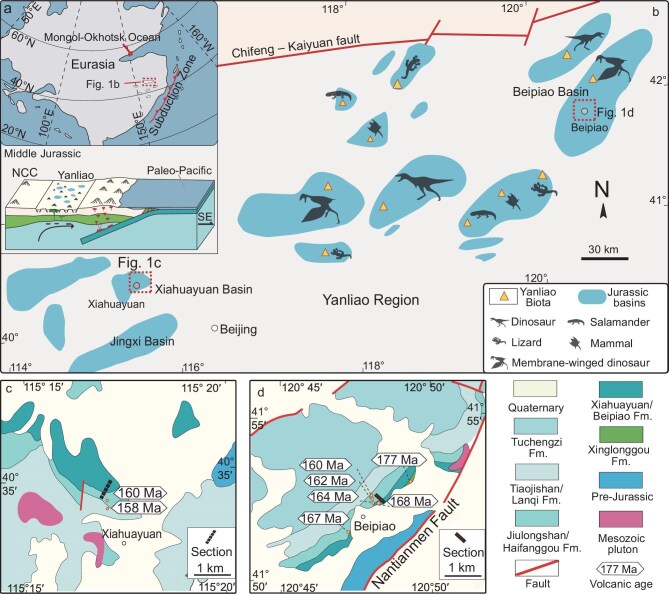
Geological setting of the Yanliao Biota. (a) Palaeogeographical setting of the North China Craton (NCC) in the late Early Jurassic (∼175 Ma) and subduction of the Palaeo-Pacific plate beneath the NCC led to the stretching and thinning of the lithosphere in the Yanliao region during Middle Jurassic (modified after [30]). (b) Distribution of the Yanliao Biota in the Yanliao region of the NCC. Geological maps of the (c) Xiahuayuan and (d) Beipiao basins in the Yanliao region (age data are from [4,13,17,37]).

Climate change, interacting with dynamic landscapes, is widely recognized as a driver of terrestrial biological diversity [9]. An illustrative example is the analysis of Neogene mammalian fossil distributions in North America and Europe, which underscores the pivotal role of environmental factors such as topography, climate and vegetation in determining diversity dynamics [10,11]. During the Early to Middle Jurassic, the subduction of the Palaeo-Pacific oceanic plate beneath the NCC positioned the Yanliao region in a back-arc tectonic setting characterized by lithospheric extension (Fig. 1) [12]. This tectonic activity generated fault-block mountains, rift valleys and extensive sedimentary basins, shaping a heterogeneous landscape [13]. Within this context, the Yanliao Biota thrived, potentially influenced by a climate shift in a warm, humid temperate zone [14–16]. Nevertheless, the mechanistic connection between climate change and the evolution of the Yanliao Biota remains an open question.

To understand the Middle Jurassic palaeoclimate and its linkage with the Yanliao Biota, we present high-resolution palynological, mudstone geochemical and organic carbon-isotope (δ^13^C_org_) records from Middle Jurassic successions in the Beipiao and Xiahuayuan basins of the Yanliao region. Integrated with lithofacies observations and climate simulation models, these data suggest a shift from wet to sub-humid conditions, associated with landscape evolution during the Bathonian global-warming interval (∼167−165 Ma). This Bathonian climate shift in the Yanliao region likely enhanced habitat diversification, contributing to the initial diversification of the Yanliao Biota.

## RESULTS AND DISCUSSION

### Stratigraphic correlations and age constraints for the sampled successions

**Figure 2.**
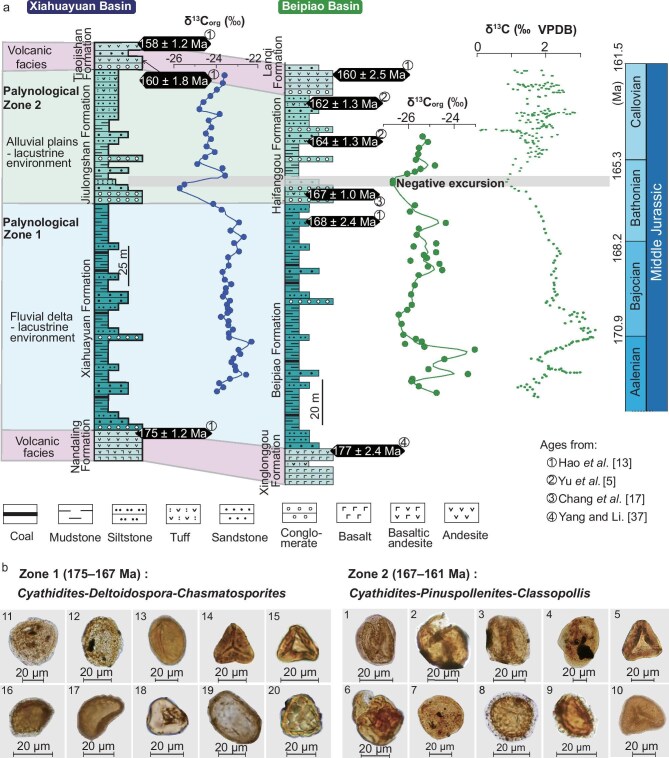
Stratigraphy of Middle Jurassic sequences in the Xiahuayuan and Beipiao basins. (a) Stratigraphic ages of the studied successions in the Xiahuayuan and Beipiao basins, along with δ^13^C_org_ values from the Xiahuayuan and Beipiao sequences. The variation in δ^13^C_carb_ values through the Middle Jurassic is based on [18]. VPDB—Vienna Peedee belemnite. Age data are from [4,13,17,37]. (b) Palynomorphs (pollen and spores) observed in the upper and lower parts of the Xiahuayuan and Beipiao successions. 1. *Pinuspollenites* sp.; 2. *Classopollis* sp.; 3. *Pinuspollenites* sp.; 4. *Pityosporites* sp.; 5. *Cyathidites minor*; 6. *Classopollis annulatus*.; 7. *Pityosporites* sp.; 8. *Lycopodiumsporites* sp.; 9. *Jiaohepollis* (undetermined genus); 10. Sinopteridaceae (undetermined genus); 11. *Chasmatosporites elegans.*; 12. *Tsugaepollenites* sp.; 13. *Monoletes* sp.; 14. *Deltoidospora* sp.; 15. *Cyathidites minor*; 16. *Lycopodiumsporites* sp.; 17. *Osmundacidites* sp.; 18. *Triletes* sp.; 19. *Laevigatosporites* sp.; 20. *Dennstaedtia* sp.

The two studied successions exhibit similar δ^13^C_org_ values, ranging from –26.0‰ to –22.6‰ in the Xiahuayuan succession and from –26.5‰ to –24.2‰ in the Beipiao succession (Fig. 2 and Table S1). The Beipiao succession displays a pronounced negative δ^13^C_org_ excursion, shifting from –24.2‰ to –26.4‰ at the boundary between the Beipiao and Haifanggou formations. Similarly, the Xiahuayuan succession shows a negative δ^13^C_org_ excursion from –23.1‰ to –25.9‰ at the boundary between the Xiahuayuan and Jiulongshan formations. The Beipiao and Xiahuayuan formations present a comparable palynological assemblage, dominated by a high proportion of pteridophyte spores (Table S2 and Supplementary Text). This assemblage, characterized by typical palynoflora from the Cyatheaceae family, including *Cyathidites* and *Deltoidospora*, is defined here as the *Cyathidites–Deltoidospora–Chasmatosporites* Zone (Fig. 2b). In contrast, the palynological assemblages of the Haifanggou and Jiulongshan formations are similar, marked by a dominance of gymnosperm pollen (Table S2 and Supplementary Text). Here, *Cyatheaceae palynoflora* decreased, while the proportion of gymnosperm pollen increased significantly, especially the bisaccate conifer pollen (*Pinuspollenites*) and monosulcate pollen (*Classopollis*). Based on these changes, this assemblage is defined as the *Cyathidites–Pinuspollenites–Classopollis* Zone (Fig. 2b).

The Beipiao and Haifanggou formations in the Beipiao basin correlate with the Xiahuayuan and Jiulongshan formations in the Xiahuayuan basin, based on the regional stratigraphic framework [13]. The negative δ^13^C_org_ excursions and palynoflora assemblages provide robust evidence for these stratigraphic correlations. A tuff layer in the upper Beipiao Formation has been dated to 168 ± 2.4 Ma [13], while tuff layers from the Haifanggou and Jiulongshan formations yielded zircon U–Pb ages of 167 ± 1.0, 164 ± 1.3, 162 ± 1.3 and 160 ± 1.8 Ma [4,13,17]. Combined with age constraints from the first and fourth units (see ‘Materials and methods’), these ages can constrain the sampled successions to ∼175−160 Ma, with the second–third unit boundary at ∼166 Ma (Fig. 2a). These age constraints might have uncertainties of 2−3 Ma due to the errors associated with the reported zircon U–Pb ages. Nonetheless, the observed negative δ^13^C_org_ excursion aligns with the late Bathonian (∼167−165.5 Ma) within this chronostratigraphic framework. A similar late Bathonian negative excursion is evident in marine carbonate carbon-isotope records [18]. We here tentatively suggested that this excursion may reflect a global carbon-cycle perturbation during the late Bathonian (LBaE, Fig. 2), thereby reinforcing the proposed chronostratigraphic framework, but more data are needed to confirm these carbon-isotope stratigraphic correlations and to understand the indicated carbon-cycle perturbation.

### The late Bathonian climate transition in the Yanliao region

**Figure 3.**
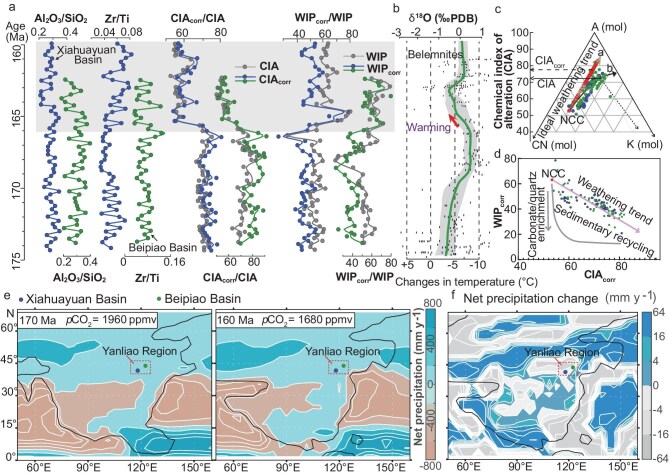
Chemical weathering indices and net precipitation simulations in the Yanliao Region. (a) Chemical weathering indices (*CIA, CIA*_corr_, *WIP, WIP*_corr_) and Al_2_O_3_/SiO_2_, Zr/Ti ratios for the Xiahuayuan and Beipiao successions. (b) δ^18^O data for the belemnites and corresponding temperature changes based on [23]. PDB—Peedee belemnite. (c) A–CN–K (Al_2_O_3_–Na_2_O + CaO*–K_2_O) diagram with a CIA scale for the analysed mudstone samples. The arrow represents the ideal weathering trend for the upper continental crust of the NCC [38]. The CIA values of the plotted samples are lower due to the K metasomatic effect. This study used the pre-metasomatized *CIA*_corr_ values (red circles) according to [12]. (d) Plot of *WIP*_corr_ versus *CIA*_corr_ [38]. The samples display a primarily linear relationship that follows the weathering trend of the upper continental crust of the NCC [39], indicating first-cycle derivation from a proximal source region in the NCC. (e) Simulated net precipitation at 170 Ma (CO_2_ = 1960 ppm) and 160 Ma (CO_2_ = 1680 ppm). (f) Change in net precipitation between 160 Ma (CO_2_ = 1680 ppm) and 170 Ma (CO_2_ = 1960 ppm).

Mudstones from the second and third sedimentary units exhibit Al_2_O_3_, SiO_2_, TiO_2_ and Zr contents in the range of 11–22 wt%, 42–72 wt%, 0.4–1.5 wt% and 154–380 ppm, respectively. Their Al/Si and Zr/Ti ratios show limited variation, ranging from 0.03 to 0.14 and 0.19 to 0.40, respectively (Table S3 and Fig. 3a). Calculated CIA (chemical index of alteration) [19] and WIP (weathering index of Parker) [20] values range from 53 to 82 and 29 to 75, respectively (Table S4; Fig. 3a). On the A−CN−K (Al_2_O_3_−Na_2_O+CaO−K_2_O) diagram (Fig. 3b), the samples follow a trend starting near the average upper-crust composition of NCC, but deviate below the ideal NCC weathering trend, indicating progressive K_2_O enrichment due to potassium metasomatism. This plot suggests a major derivation from an average provenance akin to the NCC upper crust and signifies potassium metasomatism for the studied mudstones [21]. Following the method outlined by Yang *et al.* [12], K-corrected CIA (*CIA*_corr_) and WIP (*WIP*_corr_) values range from 54 to 82 and from 21 to 79, respectively (Fig. 3a). These weathering indices display consistent trends across the successions, especially at around the second–third unit boundary, where *CIA*_corr_ presents a distinct decrease from 81 to 56 and *WIP*_corr_ shows an increase from 21 to 70. On the *CIA*_corr_ vs. *WIP*_corr_ diagram (Fig. 3c), samples generally align with the ideal weathering trend of the NCC, suggesting minimal influence from sedimentary recycling, which could cause the enrichment of quartz grains in produced sediments and thus will decrease *WIP*_corr_ (and WIP) values without influencing *CIA*_corr_ (and CIA) values [22]. Furthermore, *CIA*_corr_ values are poorly correlated with the Al/Si ratio (*r* = 0.29), an indicator of hydraulic sorting, and with the Zr/Ti and Th/Sc ratios (*r* = 0.26 and 0.08), indicators of source-rock composition (Fig. 3a and Fig. S2). Importantly, samples from each of the two studied successions plot closely within two regions on the Zr/Sc vs. Th/Sc diagram (Fig. S2) and suggest no significant change in the source-rock composition throughout the stratigraphic interval. Thus, these variations in *CIA*_corr_ (and *WIP*_corr_) values are unlikely to be related to a sorting effect or provenance changes. Consequently, the stratigraphic trends in *CIA*_corr_ and *WIP*_corr_ can be largely interpreted in terms of temporal shifts in the source chemical weathering intensity, indicating a significant reduction in the chemical weathering intensity in the source landscapes through the second–third unit transition at ∼167−165.5 Ma.

**Figure 4.**
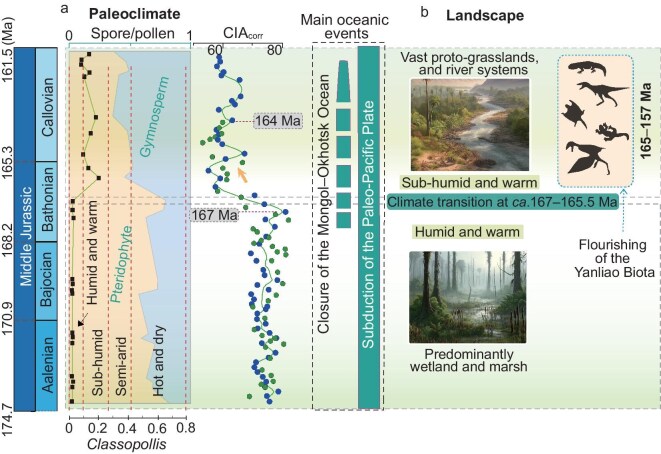
Bathonian climate and landscape changes and their linkage with the Yanliao Biota. (a) Changes in the relative proportion of pollen and spores and the abundance of *Classopollis* in the Xiahuayuan basin of the Yanliao region (this study). The line indicates the relative abundance of *Classopollis*, with the vertical bands representing different climatic regimes (e.g. sub-humid) based on the relative abundance of *Classopollis*. Also shown are *CIA*_corr_ values for the Xiahuayuan and Beipiao basins. Age data are from [4,17]. Main oceanic events documented in previous studies [13,36]. (b) Schematic representation of Middle Jurassic climatic and terrestrial landscape changes in the Yanliao region.

A high chemical weathering intensity typically corresponds to warm, humid climates, whereas lower intensity aligns with cooler or drier conditions [19]. Within the established chronostratigraphic framework, the second–third unit transition coincides with a decreasing trend in marine δ^18^O records, indicating climate warming during the late Bathonian [23,24]. Consequently, the observed decrease in the source chemical weathering intensity in the late Bathonian most likely resulted from a regional climate drying. For present-day rivers draining east China, the suspended sediments show a strong CIA–precipitation correlation (*r*^2^ = 0.83) with hydroclimate conditions [25]. Using this CIA–precipitation relationship for eastern China riverine muddy sediments, the higher CIA values of the second unit indicate precipitation of >1000 mm/yr, while the lower CIA values of the third unit should correspond to precipitation of <750 mm/yr. This interpretation is consistent with the great diminishment of coal seams from the second unit to the third unit, as observed in the logged successions (Fig. 2a). It also coincides with the palaeoflora evolution from the *Cyathidites–Deltoidospora–Chasmatosporites* Zone to the *Cyathidites*–*Pinuspollenites*–*Classopollis* Zone. The former pollen–spore zone contains flora that typically grow under warm and humid conditions [26]. The latter zone contains particularly drought-tolerant species such as *Classopollis* (10%–23%; Fig. 4a), usually denoting a sub-humid condition [27]. We further conducted palaeoclimate simulations by using the Community Earth System Model CESM 1.2.2 for 170 Ma with atmospheric *p*CO_2_ of 1960 ppmV and for 160 Ma with atmospheric *p*CO_2_ of 1680 ppmV. The modelling results show an obvious decrease in annual precipitation in the Yanliao region (Fig. 3c and d) and indicate a climate shift from humid to sub-humid from 170 to 160 Ma. These climate simulations support the advocated aridification trend recorded in the studied successions. Similar lithofacies pattern and palaeoflora replacement have been observed in other Jurassic basins within the Yanliao region, such as the Jingxi, Luanping, Lingyuan and Chaoyang basins [13,28]. Collectively, the late Bathonian warming coincided with a regional shift from humid to sub-humid conditions ∼167–165.5 Ma (Fig. 4b).

### Climate and landscape changes driving the diversification of the Yanliao Biota

Climate change significantly impacts vegetation, weathering processes and sedimentary environments, thereby influencing the regional landscape evolution [29]. Since the Early Jurassic, the Yanliao region has undergone substantial geological transformations, including the formation of fault-block mountains and rift valleys due to tectonic extension (Fig. 1e). Wu *et al*. [30] noted that the reconstructed basin patterns indicate the presence of intermontane sedimentary basins during this period (Fig. 1b). Sedimentary lithofacies from the early Middle Jurassic indicate coal-forming fluvial-delta to lacustrine environments under warm, wet climatic conditions. After the climate shifted to sub-humid conditions ∼167–165.5 Ma, sediments were deposited in a seasonally active alluvial plain environment. The reduction in precipitation led to the contraction of lakes and wetlands, and the development of alluvial sedimentary systems. This aridification trend also created conditions that were favourable for the growth of drought-resistant plants, such as cycads, low-growing shrubs and ground-cover plants, resembling early grassland-like systems (Fig. 2b). The change in vegetation cover increased soil susceptibility to erosion, potentially forming new geomorphological features.

The Yanliao Biota initially thrived in the late Bathonian within the NCC, recording significant evolutionary innovations. These include the diversification of specialized mammalian locomotor adaptations, such as swimming, arboreal lifestyles, gliding and digging behaviours [31,32], the early-branching feathered dinosaurs [7] and transitional pterosaurs, such as wukongopterids [33]. These diverse species constitute an extensive and varied group of animals that inhabited a wide array of environments, including forests, proto-grasslands, mountainous areas and subterranean habitats. The late Bathonian climate change could exert selective pressures on terrestrial vertebrates in the Yanliao region to exploit new ecological niches and undergo separate evolutionary experiments due to their developmental plasticity. Diverse habitat environments may help to drive ecomorphological diversification through ecological isolation and allopatric speciation. This is exemplified by the emergence of nearly all mammalian forms of locomotion in the Yanliao Biota [4,34] and innovations such as pennaceous feathers and four-winged morphology (e.g. *Anchiornis, Xiaotingia*) that would facilitate aerial locomotion during the dinosaur–bird transition [7,35]. These changes in the late Middle Jurassic contributed to lineage splitting in tetrapod evolution. Thus, climate change and the associated landscape evolution during the late Bathonian created variable ecological habitats, which played a crucial role in enhancing biodiversity and vertebrate evolution by providing opportunities for evolutionary innovations within major vertebrate clades of the Yanliao Biota.

## MATERIALS AND METHODS

The NCC is located in the mid-latitude regions of the Northern Hemisphere (Fig. 1a) [36] and experienced the subduction of the Palaeo-Pacific plate during the late Mesozoic [13]. The continuous westward subduction of the Palaeo-Pacific plate beneath the NCC since the Jurassic, similar to the Cenozoic eastward subduction of the Pacific plate beneath South America [1], resulted in the thickening and uplift of the continental lithosphere. This tectonic activity prompted the westward migration of the continental arc from North Korea to the Liaodong region in the eastern NCC (Fig. 1a) [12]. Located west of the continental arc, the Yanliao region underwent extensional tectonics, leading to the formation of numerous rift valleys [13]. With the tectonic extension, a series of basins developed, including the Beipiao and Xiahuayuan basins studied in this work (Fig. 1a) [30,37].

In the Beipiao and Xiahuayuan basins, the late Mesozoic sedimentary successions overlie Proterozoic to Palaeozoic sedimentary rocks (Fig. 1b). The Jurassic successions are dominated by volcano-sedimentary rocks and contain four stratigraphic units that can be correlated among the basins (Fig. 2a). The first unit is represented by the Xinglonggou Formation in the Beipiao basin and the Nandaling Formation in the Xiahuayuan basin, consisting mainly of mafic volcanic rocks dated to 177–175 Ma [13,37]. The second unit includes the Beipiao Formation in the Beipiao basin and the Xiahuayuan Formation in the Xiahuayuan basin, consisting mainly of mudstone, sandstone and conglomerate with multiple coal seams, indicative of fluvial-deltaic and lacustrine deposits (Fig. S1). The third unit comprises the Haifanggou Formation in the Beipiao basin and the Jiulongshan Formation in the Xiahuayuan basin, characterized by conglomerate, sandstone, siltstone and mudstone (Fig. 2a), representing channel and overbank deposits in an alluvial plain environment [13]. The fourth unit is represented by the Langqi Formation in the Beipiao basin and the Tiaojishan Formation in the Xiahuayuan basin, consisting of intermediate volcanic rocks, with basal andesites dated to 160–158 Ma [13]. The initial flourishing of the Yanliao Biota was recorded in the third unit (Haifanggou or Jiulongshan formations).

A total of 102 mudstone samples were collected from the second and third units, including 57 samples from the Xiahuayuan basin and 45 samples from the Beipiao basin (Fig. 1). All samples were collected from outcrops by removing the weathered surface material to a depth of 0.5–1.0 m to avoid the effects of present-day alterations. The collected mudstone samples were analysed for palynological assemblages via microprobe observation, organic carbon isotopes (${{\mathrm{\delta }}}^{13}$C_org_) by using isotope ratio mass spectrometry (IRMS) and geochemical compositions by using an X-Ray fluorescence (XRF) spectrometer. Detailed descriptions of the IRMS and XRF analytical methods are outlined in the Supplementary Material.

## Supplementary Material

nwaf194_Supplemental_File
