# Supplementary material for: Climate-change-enhanced habitat diversification for the Middle Jurassic Yanliao Biota in East Asia
Source: Natl Sci Rev. 2025 May 16;12(7):nwaf194. doi: 10.1093/nsr/nwaf194 (PMC12258141; doi:10.1093/nsr/nwaf194)
Supplement: nwaf194_Supplemental_File [file nwaf194_supplemental_file.pdf]

SUPPLEMENTAL MATERIAL for

Climate change enhanced habitat diversification for the Middle Jurassic Yanliao Biota in East Asia

Wenxing Hao[1, 2,*], Jianghai Yang[3,*], Haibing Wang[4], Ross N. Mitchell[1], Chunxia Zhang[5], Ruoyuan Qiu[1], Jiaqi Guo[6], Wang Zhang[1], Xiujuan Bao[1], Chenglong Deng[1], Xiaolin Wang[4], Yongyun Hu[6], Jin-Hui Yang[1], Guang Zhu[7], Zhonghe Zhou[4], and Rixiang Zhu[1]

[1]State Key Laboratory of Lithospheric and Environmental Coevolution, Institute of Geology and Geophysics, Chinese Academy of Sciences, Beijing 100029, China.

[2]National Key Laboratory of Deep Oil and Gas, School of Geosciences, China University of Petroleum (East China), Qingdao 266580, China.

[3]State Key Laboratory of Biogeology and Environmental Geology, School of Earth Sciences, China University of Geosciences, Wuhan 430074, China.

[4]Key Laboratory of Vertebrate Evolution and Human Origins of Chinese Academy of Sciences, Institute of Vertebrate Paleontology and Paleoanthropology, Chinese Academy of Sciences, Beijing 100044, China.

[5]Key Laboratory of Cenozoic Geology and Environment, Institute of Geology and Geophysics, Chinese Academy of Sciences, Beijing 100029, China.

[6]Laboratory for Climate and Ocean–Atmosphere Studies, Department of Atmospheric and Oceanic Sciences, School of Physics, Peking University, Beijing, China.

[7]School of Resource and Environmental Engineering, Hefei University of Technology, Hefei 230009, China.

*Corresponding authors. E-mails: wxhao10@163.com, yangjh@cug.edu.cn.

This Supplementary material contains three sections:

Supplementary Text
Figure S1-S4
Table DR1-DR3

Supplementary Text

METHODS

Palynological records

The samples were processed using standard palynological techniques. Firstly, samples weighing about 50–80 g were treated with 10% HCl and 70% HF to remove carbonates and silicates, respectively. Secondly, the residues were processed by a 10 mm nylon sieve. Finally, the residues were mounted in glycerol for identification. All the 102 samples were processed for palynological analysis, of which 45 samples were productive. The identification of palynomorph was based on Song et al. [1] and was conducted at the Geological Experiment and Testing Center, Hebei Provincial Bureau of Geology and Mineral Exploration and Development, Baoding, China. About four slides were observed for each sample and approximately 100–200 grains were counted from each sample to calculate the palynomorph abundance.

$\delta^{13}C_{org}$ values

For the $\delta^{13}C_{org}$ analyses, homogenized sample material (~0.5 g) was decarbonated in 50 mL of 10% HCl for up to 24 h. The samples were subsequently rinsed several times with distilled water to reach a neutral pH. The residues were then oven-dried at 60°C and ground in an agate mortar for further analyses. $\delta^{13}C_{org}$ analyses were undertaken at the Institute of Geology and Paleontology, Chinese Academy of Sciences, Nanjing, China. Dried samples (~2 g) were mixed with CuO and Pt, sealed in evacuated quartz tubes, and combusted at 850°C for 2 h to produce CO_2. The CO_2 was cryogenically extracted for carbon isotopic analysis using a Thermo Delta V Flash High Temperature *Plus* Elemental Analyzer. $\delta^{13}C_{org}$ values are reported in per mil (‰) relative to Vienna PeeDee Belemnite (VPDB), with precisions of better than ±0.1‰.

Major and trace elements

Samples were crushed using an agate ball mill and sieved to <200 mesh to obtain sub-samples for major and trace element analyses. The sample pretreatment for the whole-rock major element analyses involved the fusion method. The flux was a mixture of Li tetraborate, metaborate, and fluoride (45:10:5). Ammonium nitrate and Li bromide were used as an oxidant and fluxing agent, respectively. The melting

temperature was 1,050°C and the melting time was 15 min. The analyses were conducted with a ZSX Primus II wavelength-dispersive X-ray fluorescence spectrometer (Rigaku) at Wuhan SampleSolution Analytical Technology, Wuhan, China. The data were corrected with the theoretical α coefficient method. The relative standard deviation (RSD) was less than ±2%. Trace element analysis was conducted with an Agilent 7700e inductively coupled plasma mass spectrometer at Wuhan SampleSolution Analytical Technology. The detailed sample digestion procedures were as follows: (1) sample powder (200 mesh) was placed in an oven at 105°C to dry for 12 h; (2) 50 mg of sample powder was accurately weighed and placed in a Teflon bomb; (3) 1 mL of HNO_3 and 1 mL of HF were slowly added to the Teflon bomb; (4) the Teflon bomb was placed in a stainless steel pressure jacket and heated to 190°C in an oven for >24 h; (5) after cooling, the Teflon bomb was opened and placed on a hotplate at 140°C and evaporated to incipient dryness, and then 1 mL of HNO_3 was added and evaporated to dryness again; (6) 1 mL of HNO_3, 1 mL of MQ water, and 1 mL of internal standard solution were added, and the Teflon bomb was resealed and placed in an oven at 190°C for >12 h; and (7) the final solution was transferred to a polyethylene bottle and diluted to 100 g by adding 2% HNO_3.

Chemical weathering indices

Various chemical weathering indices were determined to quantify the intensity of subaerial chemical weathering. Of the widely used chemical weathering indices, Chemical Index of Alteration (CIA) [2] is based on the ratio of Al to major elements:

$$CIA = molar\ [Al_2O_3/\ (Al_2O_3 + CaO^* + Na_2O + K_2O) \times 100] \quad (1)$$

where CaO* represents the fraction of CaO in silicate minerals, which is used to avoid contributions from CaO in carbonate and phosphate minerals that are not linked to weathering processes [2]. $CaO^* = CaO - (10/3) \times P_2O_5$ (in moles) [3,4]. If $CaO^* < 5Na_2O$, then the CaO value is used as the CaO* value, and otherwise CaO* is assumed to be equivalent to Na_2O [4].

The WIP focuses on the individual mobilities of the most mobile major elements (Na, Ca, and K + Mg). A low WIP value indicates intense weathering conditions, which

is the opposite of the CIA. However, the WIP is sensitive to sedimentary recycling and sorting-induced accumulation of quartz and zircon. CIA values are not affected by these factors [5].

$$WIP = \text{molar } [(2Na_2O/0.35 + MgO/0.9 + 2K_2O/0.25 + CaO^*/0.7) \times 100] \quad (2)$$

The K-enrichment involves the addition of K_2O to aluminous clays, thereby forming authigenic illite, which results in samples plotting toward the K apex of the A–CN–K triangle (Fig. 3b). This enrichment results in lower CIA and higher WIP values. Therefore, a correction of the potassium metasomatism of some samples that deviate from the weathering trend is needed. Panahi et al. [6] proposed an alternative way of evaluating the amount of added diagenetic K_2O and correcting the CIA and WIP:

$$K_2O^* = [m \times A + m \times (C^* + N)]/(1 - m) \quad (3)$$

Here we choose NCC upper crust as the protolith, and calculate m as:

$$m = K/(A + (C^* + N) + K) \text{ for the protolith} \quad (4)$$

K, A and (C* + N) represent the molar proportions of K_2O, Al_2O_3 and (CaO^* + Na_2O) of the NCC, respectively. Therefore, a corrected CIA_{corr} and WIP_{corr} based on the specific composition of their provenances can be calculated.

$$CIA_{corr} = \text{molar } [Al_2O_3/(Al_2O_3 + CaO^* + Na_2O + K_2O^*) \times 100] \quad (5)$$

$$WIP_{corr} = \text{molar } [(2Na_2O/0.35 + MgO/0.9 + 2K_2O^*/0.25 + CaO^*/0.7) \times 100] \quad (6)$$

A third chemical weathering index, τ_{Na}, denotes the chemical depletion of sodium in weathering materials relative to the fresh parent rocks, with positive values showing retention and negative values indicating Na loss during weathering, using Zr as an immobile element:

$$\tau_{Na} = (Na/Zr)_{mudstone}/(Na/Zr)_{protolith} - 1 \quad (7)$$

where $(Na/Zr)_{mudstone}$ represent the concentrations of Na and Zr in the analyzed mudstones, and $(Na/Zr)_{protolith}$ mean the concentrations of Na and Zr in the average source rock, we here selected the upper crust of the NCC [7] as the protolith, using its composition as the average source rock.

Climate model and experimental design

CESM1.2.2 (Community Earth System Model, version 1.2.2), developed by the National Center for Atmospheric Research (NCAR), is a high-resolution model designed to simulate interactions within the Earth's climate system. It includes subsystems for the atmosphere, ocean, sea ice, land, and ice sheets, all integrated through the CPL7 coupling framework. CESM1.2.2 is commonly used for both modern and paleoclimate studies, including Phanerozoic climate research dating back to the Cambrian (~540 Ma) [8]. For this study, we used Community Atmosphere Model version 4 (CAM4) for the atmosphere with a resolution of 3.75° in both longitude and latitude and 26 vertical layers. The land component, Community Land Model version 4 (CLM4), shares the same horizontal resolution and simulates natural vegetation using a dynamic carbon-nitrogen model. River transport is modeled at 0.5° × 0.5°, directing runoff to the ocean. The ocean component runs on a 3° irregular grid with 60 vertical layers, while sea ice is modeled using CICE4 on the same grid. Following the study by Li et al. [9], we set CO_2 concentrations at six times (1680 ppm) and seven times (1960 ppm) pre-industrial levels (280 ppm) for the 170 Ma and 160 Ma periods, respectively, to align with global average temperature estimates reported by Scotese et al. [10]. Net precipitation is the difference between precipitation and evaporation (P–E). Additionally, to evaluate CO_2 sensitivity and investigate the potential impact of warming, we conducted slice experiments for both periods using a ten times pre-industrial CO_2 concentration (2800 ppm)

RESULTS

Palynological assemblages

The collected palynomorphs are represented by pteridophyte spores and gymnosperm pollen. They include 29 spore species and 36 pollen taxa. The relative abundance of all palynomorphs within the succession was calculated, and their distribution was noted (Table DR1).–Based on variations in the abundance of the dominant taxa in the stratigraphy analysis, two pollen and spore zones were identified (Table DR1).

Zone 1: *Cyathidites–Deltoidospora–Chasmatosporites*. This zone belongs to the lower parts of the sampled Xiahuayuan and Beipiao successions (the Xiahuayuan and

Beipiao formations, respectively). It is characterized by a high proportion of pteridophyte spores (65%) and gymnosperm pollen (35%). The dominant pteridophyte spores are from the Cyatheaceae (54%), including *Cyathidites* (42%) and *Deltoidospora* (12%). Additionally, *Lycopodiumsporites* (4%), *Laevigatosporites* (3%), and *Osmundacidites* (1%) are common. Other spore types, such as *Monoletes* and *Triletes,* occur occasionally. The gymnosperm pollen represents only 35% of the total assemblage. The dominant pollen group is monosulcate pollen, accounting for 23% of the total, including *Chasmatosporites* (11%) and *Cycadopites* (10%). Other pollen types such as *Classopollis, Taeniaesporites,* are also present but occur in low abundance. The bisaccate conifer pollen is common, accounting for an average of 12% of the total, with *Pinuspollenites* (6%), *Pityosporites* (3%), and *Podocarpidites* (2%) dominating the assemblage.

Zone 2: *Cyathidites–Pinuspollenites–Classopollis*. This zone belongs to the upper parts of the sampled Xiahuayuan and Beipiao successions (Jiulongshan and Haifanggou formations, respectively). It is dominated by pteridophyte spores (47%) and gymnosperm pollen (53%). The dominant pteridophyte spores are from the Cyatheaceae, including *Cyathidites* (37%) and *Lycopodiumsporites* (8%). Other spore types such as *Deltoidospora* and *Triletes* are not abundant. The gymnosperm pollen represents 53% of the total assemblage. The dominant pollen group is bisaccate conifer pollen, accounting for 29% of the total, including *Pinuspollenites* (23%), *Pityosporites* (4%) and *Abies* (2%). Other pollen types such as *Protoconiferus*, *Tsugaepollenites*, and *Podocarpidites* are rare. The monosulcate pollen (24%), such as *Classopollis* (12%), becomes the most abundant element. Other types, including *Chasmatosporites* (5%) and *Cycadopites* (4%), are also present. Pollen types like *Jiaohepollis* and others are relatively rare.

Simulation of terrestrial environmental evolution

Figs. 3A and 3B show the distributions of the annual mean net precipitation (defined as precipitation minus evaporation, P–E) at 170 Ma and 160 Ma in the Yanliao

Region of the East Asian continent. To account for potential warming effects, we conducted additional CO_2 sensitivity experiments, including comparisions at 10 times the pre-industrial CO_2 concentration (Fig. S4). The results indicate that even under elevated CO_2 levels, the net precipitation in the Yanliao Region decreased from the Middle Jurassic at 170 Ma (Zone 1) to 160 Ma (Zone 2; Fig. 3C).

Mudstone geochemical compositions and weathering index values

Mudstones samples have variable contents of SiO_2 (42–72 wt.%), Al_2O_3 (11–22 wt.%), MgO (0.65–2.78 wt.%), Na_2O (0.64–4.9 wt.%), K_2O (2–6 wt.%), Fe_2O_{3T} (2–22 wt.%), CaO (0.37–7.32 wt.%), and TiO_2 (0.4–1.5 wt.%) (Table DR2). The Zr/Sc ratios vary from 11–29 for most samples, but are higher (> 30) in a few samples from the Beipiao succession. They have Th/Sc ratios in the range of 0.4–2.7 (Table DR3) and plot within a small region in the Th/Sc vs. Zr/Sc diagram (Fig. 3D). The Zr/Ti ratio also has a limited variation in the range of 0.3–0.9. The intensity of chemical weathering in mudstone sources was quantified using index values of the Weathering Index of Parker (WIP) [11], the Chemical Index of Alteration (CIA) [2], and τ_{Na} [12]. To reduce the influence of potassium metasomatism, we here selected the upper crust of the NCC as the protolith, using its composition as the average source rock, to calculate the CIA_{corr}, WIP_{corr}, and τ_{Na} values as per the methodology proposed by Yang et al. [5]. In the Xiahuayuan succession, calculated values are 54 to 81 for CIA_{corr}, 29 to 74 for WIP_{corr}, and –0.83 to –0.11 for τ_{Na}. In the Beipiao succession (Fig. 3), the values are 53 to 82 for CIA_{corr}, 32 to 72 for WIP_{corr}, and –0.89 to –0.09 for τ_{Na}. There is a large decrease in CIA_{corr} and increase in WIP_{corr} and τ_{Na} at the boundaries between the Xiahuayuan and Jiulonggou formations, and between Beipiao and Haifanggou formations. Throughout the sampled successions, the CIA_{corr} values are strongly inversely correlated with WIP_{corr} ($r = 0.79$) and τ_{Na} ($r = 0.77$) (Fig. S3), denoting consistent weathering trends. Sediment provenance, sedimentary recycling, and hydraulic sorting are significant non-weathering factors that affect weathering indices of resultant sedimentary records [2,5,12]. For the analyzed samples, their CIA_{corr} values show poor correlations with

Al_2O_3/SiO_2, Zr/Ti and Th/Sc ratios (r = 0.29, 0.08, 0.26 respectively; Fig. S2 and Table DR2), which are proxies for dynamic sorting, sedimentary recycling, and provenance change, respectively [3,13,14]. In addition, these samples plot mainly along the ideal weathering trend of the NCC on the A-CN-K (Fig. 3B) and WIP_{corr}-CIA_{corr} diagrams (Fig. 3C), indicating a persistent sedimentary source with an average composition comparable to the NCC [3,15]. All these observations, along with the Zr/Ti, Th/Sc and Zr/Sc ratios, suggest little or no influences on the compositional trends from hydraulic sorting, sedimentary recycling, and provenance change [16,17].

Mudstone $\delta^{13}C_{org}$ trend

The two studied successions exhibit similar $\delta^{13}C_{org}$ values, which range from –26.0‰ to –22.6‰ for the Xiahuayuan succession and from –26.5‰ to –24.2‰ for the Beipiao succession (Fig. 3; Table DR4). The Beipiao succession shows an obvious $\delta^{13}C_{org}$ negative excursion from –26.4‰ to –24.2‰ at the boundary between the Beipiao and Haifanggou formations. For the Xiahuayuan succession, a similar $\delta^{13}C_{org}$ negative excursion from –25.9‰ to –23.1‰ is observed at the boundary between the Xiahuayuan and Jiulongshan formations.

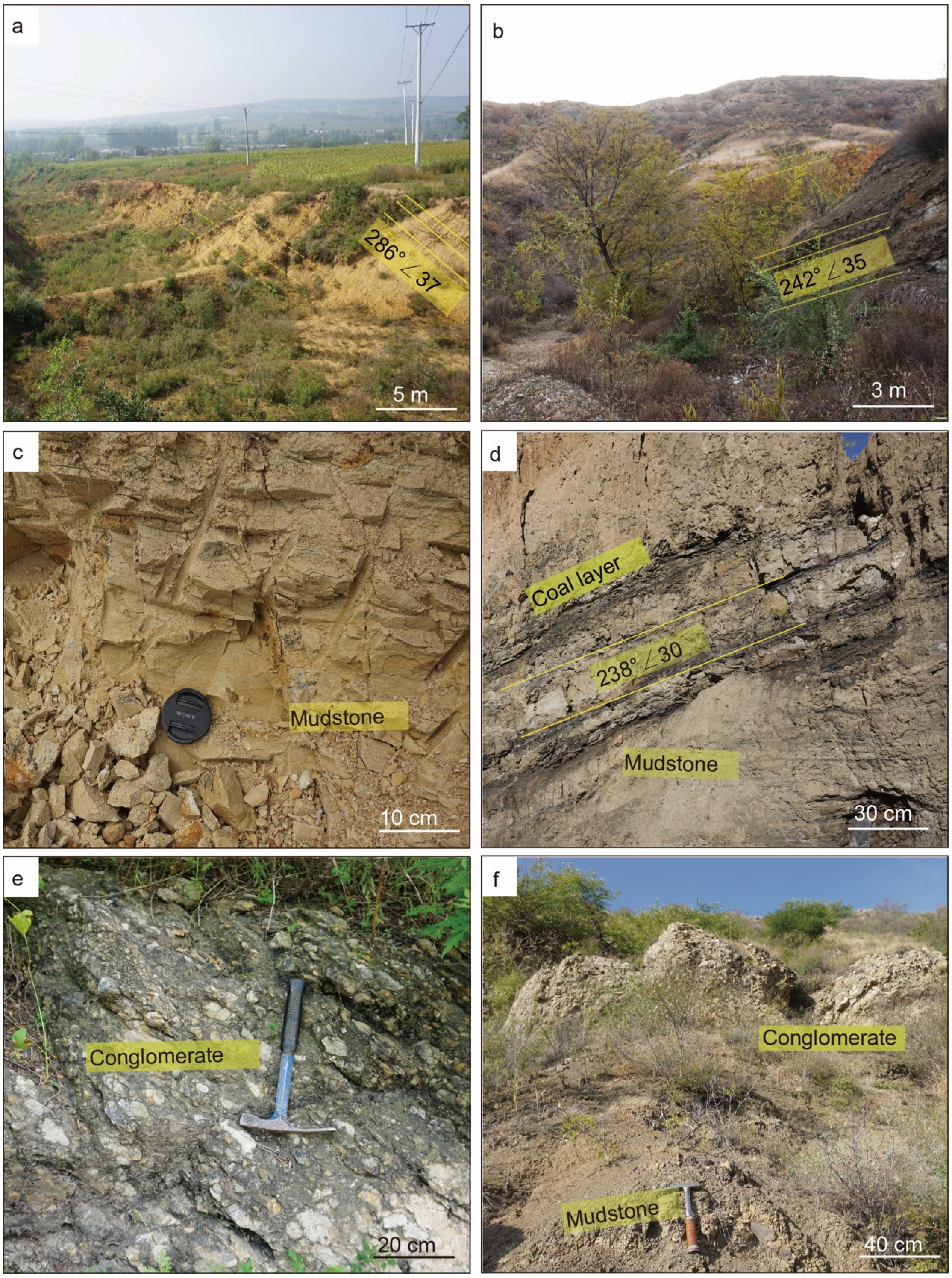

Figure S1. The field photographs of the Beipiao (41°50'35.83"N; 120°47'34.58"E) (a) and Xiahuayuan (40°33'33.88"N; 115°17'35.32"E) (b) successions. c Mudstone in the Haifanggou formation in the Beipiao basin. d Mudstone and coal layer in the Beipiao formation in the Beipiao basin. Conglomerate in the Haifangou (e) and Jiulongshan (f) formations.

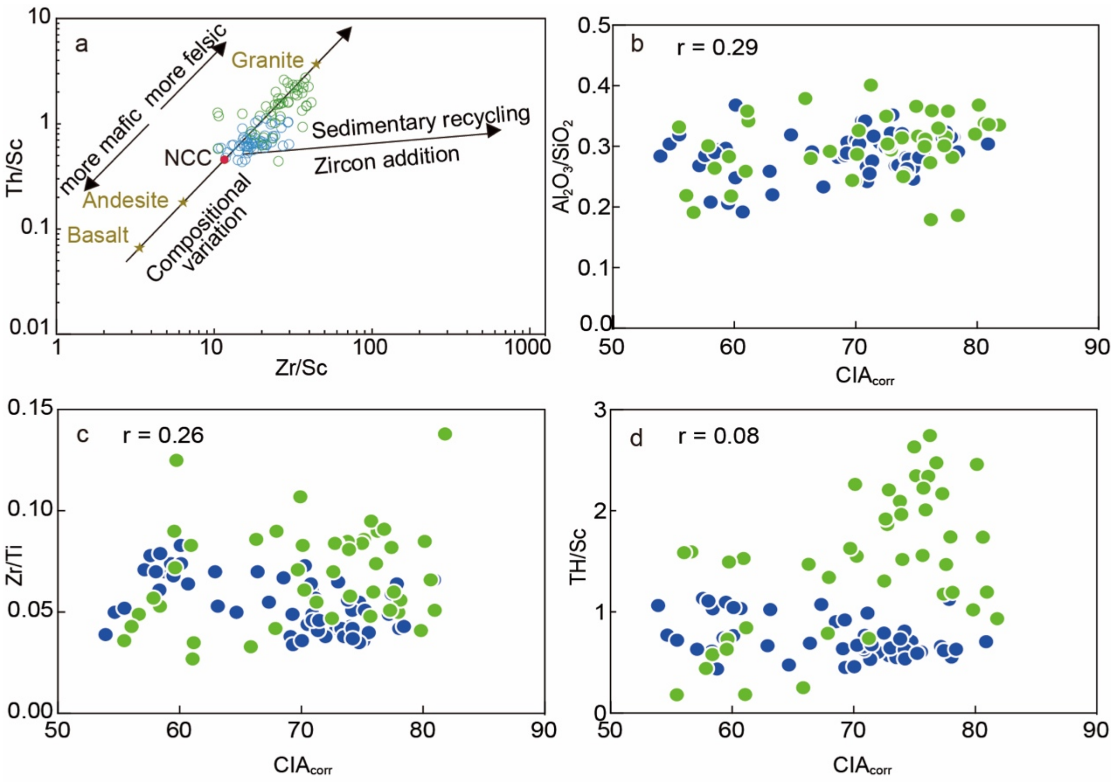

Figure S2. (a) Plot of Zr/Sc versus Th/Sc [4] for the analyzed mudstone samples compared to average ratios for typical source rocks and the predicted zircon enrichment trend due to sediment recycling. CIA_{corr} values show non-existent correlations with Al_2O_3/SiO_2 (b), Zr/Ti (c) and Th/Sc (d) ratios.

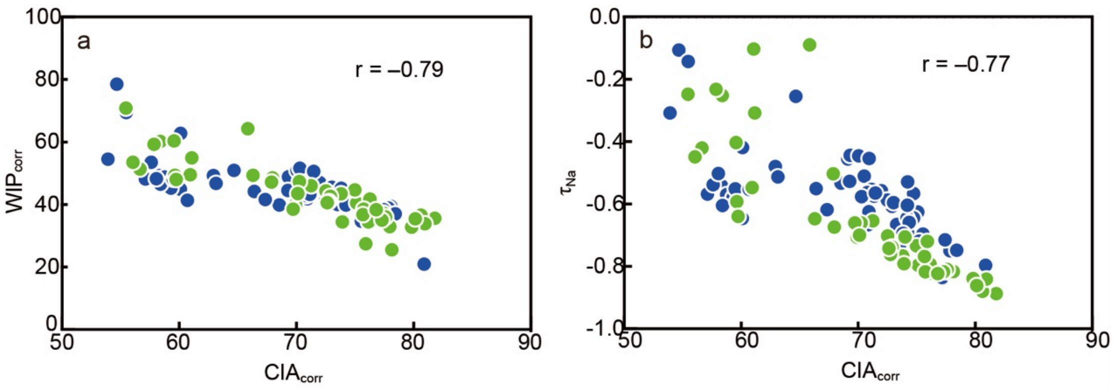

Figure S3. CIA$_{corr}$ values are strongly inversely correlated with WIP$_{corr}$ (a) (r = –0.79) and τNa (b) (r = –0.77), denoting consistent weathering trends.

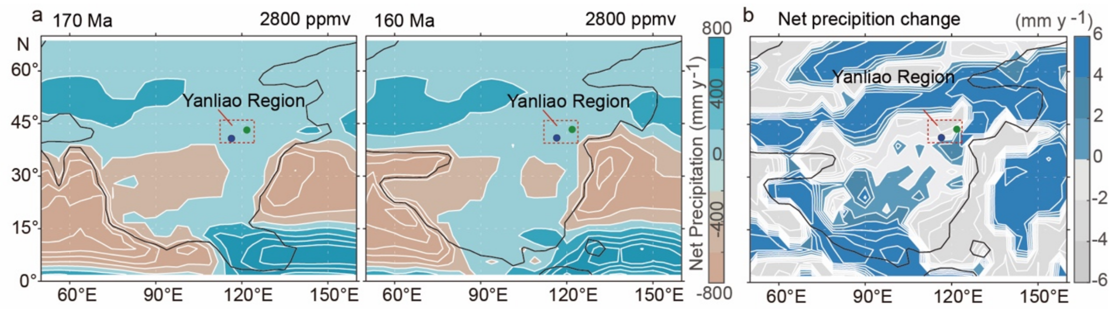

Figure S4. Net precipitation changes in the Yanliao Region from the Zone 1 to Zone 2. (a) Net precipitation results at 170 Ma with 2800 ppm CO_2 concentration. (b) Net precipitation results at 170 Ma with 2800 ppm CO_2 concentration. (c) Change in net precipitation between 160 Ma (CO_2 at 2800 ppm) and 170 Ma (CO_2 at 2800 ppm).

REFERENCES CITED

1. Song ZC, Shang YK, Liu ZS *et al*. *Fossil Spores and Pollen of China (II): The Mesozoic Spores and Pollen*. 2000, 1–710.
2. Nesbitt H, Young G. Early Proterozoic climates and plate motions inferred from major element chemistry of lutites. *Nature* 1982; **299**: 715–7.
3. Fedo CM, Nesbitt HW, Young GM. Unraveling the effects of potassium metasomatism in sedimentary rocks and paleosols, with implications for paleoweathering conditions and provenance. *Geology* 1995; **23**: 921–4.
4. McLennan SM. Weathering and global denudation. *J Geol* 1993; **101**: 295–303.
5. Yang JH, Cawood PA, Du YS *et al*. Early Wuchiapingian cooling linked to Emeishan basaltic weathering? *Earth Planet Sci Lett* 2018; **492**: 102–11.
6. Panahi A, Young GM, Rainbird RH. Behavior of major and trace elements (including REE) during Paleoproterozoic pedogenesis and diagenetic alteration of an Archean granite near Ville Marie, Québec, Canada. *Geochim Cosmochim Acta* 2000; **64**: 2199–220.
7. Gao S, Luo TC, Zhang BR *et al*. Chemical composition of the continental crust as revealed by studies in East China. *Geochim Cosmochim Acta* 1998; **62**: 1959–75.

8. Li X, Hu YY, Guo JQ *et al*. A high-resolution climate simulation dataset for the past 540 million years. *Sci Data* 2022; **9**: 371.

9. Li X, Hu YY, Yang J *et al*. Climate variations in the past 250 million years and contributing factors. *Paleoceanogr Paleoclimatol* 2023; **38**: e2022PA004503.

10. Scotese CR, Wright N. PALEOMAP Paleodigital Elevation Models (PaleoDEMS) for the Phanerozoic PALEOMAP Project, 2018.

11. Parker A. An index of weathering for silicate rocks. *Geol Mag* 1970; **107**: 501–4.

12. Rasmussen C, Brantley S, Richter DD *et al*. Strong climate and tectonic control on plagioclase weathering in granitic terrain. *Earth Planet Sci Lett* 2011; **301**: 521–30.

13. Johnsson MJ. The system controlling the composition of clastic sediments. In: Johnsson MJ and Basu A (eds). *Processes Controlling the Composition of Clastic Sediments*. Special Paper of the Geological Society of America, 1993, **284**: 1–19.

14. Garzanti E, Resentini A. Provenance control on chemical indices of weathering (Taiwan river sands). *Sediment Geol* 2016; **236**: 81–95.

15. Yang JH, Cawood PA, Du YS *et al*. Reconstructing Early Permian tropical climates from chemical weathering indices. *Geol Soc Am Bull* 2016; **128**: 739–51.

16. Garzanti E, Padoan M, Setti M *et al*. Weathering geochemistry and Sr-Nd fingerprints of equatorial upper Nile and Congo muds. *Geochem Geophys Geosyst* 2013; **14**: 292–316.

17. Dinis P, Garzanti E, Vermeesch P *et al*. Climatic zonation and weathering control on sediment composition (Angola). *Chem Geol* 2017; **467**: 110–21.

Table DR1.

Statistics of spores and pollen from Xiahuayuan and Beipiao successions.

Sample	xhy01	xhy02	xhy03	xhy05	xhy06	xhy11	xhy13	xhy18	xhy20	xhy21	xhy22	xhy23	xhy24	xhy32	xhy33	xhy34	xhy35	xhy44	xhy45	xhy46	xhy52	xhy53
Height (m)	142.10	140.03	138.15	134.26	132.77	116.22	110.06	94.90	89.10	86.22	82.03	78.14	75.12	50.11	48.19	46.02	44.12	28.10	26.23	23.88	10.11	8.21
Deltoidospora		1		1		1				2	15	22	13	23	12	30	30	15	26	12	18	26
D.microlepioidites							1				2											
D.gradata													1		2	1					1	
D.magna									1				1				2		1			1
D.regularis											1							1		1		
Cyathidites		12	7	6	14		12	9	12	8	12	17	18	20	18	28	11	15	22	17	18	20
C.minor	20	19	18	18	22	20	27	28	18	24	70	73	68	67	60	64	48	58	65	40	65	59
C.australis	2				4						12	2	1	12	8	8	2	10			10	
C.triangularis	2		1								1	3	10	12	13					10		3
C.medicus				5	1		2												2		3	
Lycopodiumsporites	2	4	12	8	7	4	4	9	5	7	3	1	3	2	2	8	1	18	8	3	12	10
L.austroclavatidites		1											1	4	2		5			3		
L.semimuris						1		1				1		2	2							
L.paniculatoides			1								1											
L.subrotundum	1								1						1				1			
Osmundacidites			1			1					1	2		3	2	1	3	2			3	1
O.parvus									1		1	1			3		3			1		
O.diversispinulatus					2		1						2									
O.elegans													2	1							1	
O.wellmanii											2	1							1			
Monoletes						2			3					6	6		1					1
Triletes	2		3		2										1					3	1	

Asseretospora gyrata																		2				
Laevigatosporites		1		1			2	1			9		11	4	8	6	9	3	1	6	2	8
Verrucosisporites												4			3	3			2			1
Raistrickia									1			1					1					
Sphagnumsporites															1							
S.psilatus							1															
S.minor				1														1				
Pityosporites	3	3	2	3	4	4	3	1	4	2	5		16		10			22		1		13
P. microalatus								1				1	2	3		2		1		3		
P. minimus			1													3						
Pinuspollenites	25	27	22	15	44	14	22	22	10	18	4	3	8	13	4	5	20	18	14	6	12	8
Podocarpidites											8	2		5	3		9	4	11		8	
Protoconiferus			2			2							3			1	3			1		3
Abies	3	5	2		2	2	1	2	3	2			1				3			2		
Tsugaepollenites									1						1						1	
Cycadopites	2	3	5	5	1	6	4	2	2	4		20	40	10	2	20	25		33		28	14
C.carpentieri			1								1											
C.deterius		1					1	1						2		1	2	2		1		
C.typicus				1						1									1			
C.nitidus															1							
Chasmatosporites	3	3	3	4	6	7	5	4	2	2	12	8	4	9	17	11	12	13	20	9	20	15
C.apertus				2						2					5	3	5		4	11	1	
C.minor	1							2							2	2	1				2	
C.elegans			1			1					1			3		2	1				2	1
C.hians					1				1					3		5	1	4	2		1	2

Sinopteridaceae																						
(undetermined genus)	1		2		1									1	1							
Jiaohepollis							3							4	2		3			4		
Taeniaesporites		2			2							1		2	3			1		1		
Classopollis	6	3	5	9	11	13	13	2	8	18									1			1
C.minor	1	2	1		1		2	1					2									
C.annulatus			1	2				3		1	1				1							
C.triangulus	3	1							1	1												
C.classoides		1				2		2	1													
Concavisporites			1																			
C.asper												1										
C.intrastriatus					1			1		1								1				
C.bohemiensis		1																				
Leptolepidites										1				1					1			
Baculatisporites	1					1					1											
Conbaculatisporites		1												1					1			
Concentrisporites															1							
C.minor																						
C.fragilis					1												1				1	

Sample	xhy54	xhy57	BP02	BP03	BP08	BP15	BP17	BP18	BP22	BP25	BP26	BP28	BP29	BP30	BP32	BP33	BP34	BP35	BP37	BP38	BP40	BP41	BP42
Height (m)	5.88	0.00	94.24	91.98	90.22	66.21	64.17	58.04	53.21	50.22	48.04	40.03	35.88	34.13	28.12	24.05	22.13	21.21	19.21	18.22	10.05	8.13	6.22
Deltoidospora	24	24	1		2	17	22	23	17	33	18	16	27	13	36	19	23	21	35	33	24	22	15
D.microlepioidites	1									1					1								1
D.gradata					1	1								1					2	1			
D.magna											2						1	1				2	

D.regularis								1								1							
Cyathidites	27	18	21	13	6	31	20	10	24	18	27	23	23	10	20	13	19	18	14	20	20	23	18
C.minor	68	74	25	28	23	33	48	55	50	48	44	53	42	53	47	38	58	42	42	33	44	47	54
C.australis		4	3		5			4															
C.triangularis		1				6					3			2	1	2	3		3		6	1	
C.medicus	4	8							2			6	1					2		1			1
Lycopodiumsporites	4	4	2	8	9	8	3	6	7	4		11		8		8	7	6	4	7	12	3	6
L.austroclavatidites							6	5				1			4				5				
L.semimuris											2		8				4					2	
L.paniculatoides			3																				
L.subrotundum				1				1					1										
Osmundacidites	2	3	1			4	1	1	1	2	1	1	1	5		3	6	4	1	1	2		3
O.parvus																							
O.diversispinulatus		1							1			1									1		
O.elegans						1																	
O.wellmanii																		1					
Monoletes		8						2				1		7	1						9		
Triletes			1			4					1				5				1			6	
Asseretospora gyrata						1			4						4		6			5			
Laevigatosporites	10	12	1				9	13	1	14		12		6	16	1	5	6	8	3	9	1	4
Verrucosisporites									1			3		4		1		3				1	
Raistrickia						1			1				1				1						1
Sphagnumsporites																							
S.psilatus		1															1					1	
S.minor										1													
Pityosporites	2		1	5	6	15	8	20		9	3		4	4	12	7	4		18	1		1	1

P. microalatus									1													1	
P. minimus				1								1			1				2				
Pinuspollenites	12	7	22	15	15	5	8	14	15	18	6	11	3	7	21	10	20	15	20	13	18	22	13
Podocarpidites	12	4				6		11	4	1		7		1		4	7	8	6		1	1	8
Protoconiferus								4					3			1			1		1		
Abies		3	3	1			2				1		1		1		1		1				
Tsugaepollenites					1																		
Cycadopites	17		4	2	5	17		33	28	15	2	43		22	16	15	18	8	30	28	18	32	26
C.carpentieri						1																	
C.deterius				1			1				1	2	2		1						1		
C.typicus		1	1											1				2					
C.nitidus								2					3										
Chasmatosporites	16	15	1	2	2	12	2	2	2	20	11	12	13	20	34	20	15	26	17	33	28	22	30
C.apertus		1				2	2	2	5	5	3	5		4	1		1		1				
C.minor		1		2								1	2		2	2	4	2	1	2			
C.elegans			1				5					1			3	2			4			10	
C.hians	1	1					2	2	3		5	1	4	2		1	2	1	1				
Sinopteridaceae (undetermined genus)				1																			
Jiaohepollis	3					1			2			2			4				2				3
Taeniaesporites						1											4				1		
Classopollis			3	8	6				2		1					1			1		1		
C.minor				2					1														
C.annulatus			3									1						1					
C.triangulus					2																	1	
C.classoides																							
Concavisporites																							

C.asper								3													1	
C.intrastriatus																		2				
C.bohemiensis					1										3							
Leptolepidites							1												1			
Baculatisporites	1										2				2							1
Conbaculatisporites																				2		
Concentrisporites											1							1		1		
C.minor						1			1										1			
C.fragilis											1			`								

Table DR2.

Major element contents (%), ratios and weathering index values of the analyzed mudstones from Xiahuayuan and Beipiao successions.

Sample	SiO2	Al2O3	Fe2O3T	CaO	MgO	Na2O	K2O	TiO2	MnO	P2O5	LOI	Total	CIA	CIA*	CIX	WIP	WIP*	τNa
XHY01	68.532	13.309	4.229	2.166	1.994	2.189	3.267	0.685	0.046	0.177	3.442	100.0	53.9	56.3	65.1	59.5	51.0	-0.61
XHY02	68.446	13.361	4.228	2.158	2.026	2.13	3.275	0.684	0.043	0.179	3.451	100.0	54.1	56.7	65.4	59.1	50.5	-0.61
XHY03	69.795	12.584	3.582	2.309	1.867	2.172	3.094	0.688	0.043	0.174	3.147	99.5	54.5	57.1	64.5	56.5	48.2	-0.63
XHY04	66.219	12.679	4.148	3.494	2.132	1.89	3.513	0.624	0.061	0.188	4.449	99.4	55.8	60.1	64.7	57.6	44.9	-0.65
XHY05	64.118	12.608	3.529	5.284	1.945	1.956	3.572	0.611	0.113	0.175	5.888	99.8	55.0	59.3	64.0	58.3	45.3	-0.65
XHY06	72.406	12.769	3.151	0.574	2.053	1.951	2.973	0.609	0.021	0.14	2.783	99.4	54.4	56.5	66.5	54.9	48.1	-0.63
XHY07	67.876	13.623	5.606	0.864	2.07	2.171	3.186	0.67	0.034	0.164	3.046	99.3	54.7	56.9	66.0	58.8	51.3	-0.60
XHY08	67.284	14.323	4.969	1.527	1.845	2.464	3.413	0.659	0.032	0.322	3.175	100.0	54.4	56.7	64.9	62.8	54.4	-0.58
XHY09	66.187	15.041	5.365	1.104	2.08	2.615	3.486	0.679	0.027	0.217	3.066	99.9	54.9	57.1	65.0	65.5	57.4	-0.56
XHY10	68.11	12.873	2.748	3.9	1.866	2.587	2.788	0.493	0.075	0.161	4.504	100.1	52.7	53.9	63.9	58.7	54.5	-0.58
XHY11	64.473	14.179	6.302	2.089	2.775	1.803	3.639	0.675	0.044	0.319	3.909	100.2	58.9	63.2	67.2	59.5	46.8	-0.64
XHY12	65.16	14.031	3.29	4.056	2.288	2.365	3.077	0.637	0.077	0.181	4.92	100.1	55.8	57.6	66.0	59.8	53.5	-0.59
XHY13	65.205	12.415	3.203	5.081	2.139	2.013	2.925	0.623	0.083	0.177	5.75	99.6	55.9	58.4	65.7	54.0	46.5	-0.64
XHY14	66.024	12.895	3.103	4.559	2.011	2.128	3.019	0.593	0.072	0.169	5.221	99.8	55.6	58.1	65.6	55.8	48.3	-0.63
XHY15	65.226	13.432	3.045	4.682	2.117	2.063	3.179	0.661	0.086	0.174	5.411	100.1	56.7	59.5	66.2	56.7	48.1	-0.63
XHY16	62.605	14.579	5.969	2.666	2.56	1.462	4.439	0.68	0.048	0.165	4.622	99.8	60.2	67.4	66.9	61.7	41.6	-0.68
XHY17	62.064	11.903	3.435	7.324	1.974	1.722	3.07	0.582	0.139	0.205	7.619	100.0	57.0	60.7	65.9	51.4	41.4	-0.68
XHY18	65.586	15.707	5.017	1.357	2.535	4.909	2.306	0.634	0.042	0.216	2.168	100.5	55.6	54.7	59.8	74.6	78.5	-0.39
XHY19	62.02	17.678	5.118	2.347	2.143	2.995	3.881	0.679	0.047	0.228	2.985	100.1	57.9	60.1	65.9	71.8	62.8	-0.52
XHY20	65.48	15.461	4.808	1.964	2.273	3.917	2.568	0.639	0.033	0.216	2.256	99.6	55.7	55.5	62.6	68.6	69.5	-0.46
XHY21	65.05	16.427	5.083	0.683	1.772	2.352	2.808	1.069	0.01	0.283	4.12	99.7	68.7	70.5	70.4	51.3	46.4	-0.64
XHY22	60.733	17.491	6.775	0.803	2.108	1.958	3.121	1.143	0.009	0.284	5.225	99.7	70.3	72.9	72.6	51.6	44.6	-0.66

XHY23	41.858	10.789	32.692	0.704	1.414	0.648	2.194	0.669	0.445	0.497	8.534	100.4	75.3	80.9	75.8	28.7	20.9	-0.84
XHY24	60.633	18.356	5.844	0.978	1.715	1.801	3.653	1.2	0.029	0.371	5.463	100.0	70.1	74.0	72.6	53.7	42.9	-0.67
XHY25	53.51	18.337	6.661	2.333	2.148	1.012	3.665	1.221	0.026	1.24	9.291	99.4	72.6	77.2	76.5	48.3	36.5	-0.72
XHY26	57.965	17.708	7.108	1.14	2.166	1.493	3.312	1.154	0.051	0.365	7.585	100.0	70.9	74.2	74.5	49.6	41.0	-0.68
XHY27	59.548	18.201	5.977	1.022	2.135	2.053	3.203	1.177	0.012	0.332	6.327	100.0	69.7	72.0	72.6	53.6	47.0	-0.64
XHY28	60.888	17.324	6.452	1.231	1.752	2.365	3.209	1.103	0.078	0.42	4.973	99.8	66.8	69.1	70.1	55.7	48.9	-0.62
XHY29	62.018	16.515	6.642	1.093	1.736	2.026	2.899	1.076	0.019	0.409	5.347	99.8	68.8	70.9	71.8	49.6	43.9	-0.66
XHY30	60.578	17.574	5.216	0.883	1.876	1.672	3.311	1.145	0.012	0.191	7.346	99.8	70.1	73.3	73.5	50.4	41.8	-0.68
XHY31	55.46	17.735	5.792	1.293	2.042	1.336	3.374	1.277	0.012	0.284	11.107	99.7	70.2	73.5	75.2	49.0	40.1	-0.69
XHY32	57.609	20.497	4.524	1	1.924	1.215	4.223	1.507	0.011	0.333	6.976	99.8	72.9	78.1	75.7	53.9	39.4	-0.70
XHY33	62.388	17.2	5.597	0.973	1.658	1.985	3.326	1.107	0.011	0.272	5.105	99.6	68.3	71.4	71.4	52.8	44.2	-0.66
XHY34	56.226	17.83	8.425	0.804	2.435	1.435	3.127	1.047	0.027	0.161	8.269	99.8	72.3	75.1	75.6	48.1	40.9	-0.68
XHY35	58.077	17.812	7.633	1.627	1.895	2.212	3.07	1.12	0.015	0.585	5.821	99.9	67.6	69.3	71.9	54.0	48.8	-0.62
XHY36	60.357	18.732	5.57	1.07	1.786	2.436	3.342	1.175	0.009	0.308	4.92	99.7	67.9	70.1	71.0	57.6	51.0	-0.61
XHY37	64.545	16.076	5.63	0.942	1.523	2.13	2.802	0.895	0.097	0.212	4.848	99.7	67.5	69.3	71.1	49.4	44.5	-0.66
XHY38	59.181	17.023	8.399	0.612	2.314	1.508	2.916	0.915	0.025	0.091	6.723	99.7	72.2	74.7	75.1	46.4	40.2	-0.69
XHY39	60.685	17.109	7.435	0.575	2.086	1.593	2.942	0.94	0.022	0.051	6.509	99.9	71.7	74.2	74.6	46.8	40.6	-0.69
XHY40	63.721	15.411	6.819	0.824	1.869	1.918	2.73	0.862	0.042	0.255	5.409	99.9	68.7	71.0	71.6	47.4	41.9	-0.68
XHY41	59.551	17.435	6.9	1.718	1.717	1.666	3.469	1.102	0.029	0.527	5.788	99.9	67.6	70.9	72.8	52.3	42.9	-0.67
XHY42	62.074	17.328	5.723	0.856	1.564	1.68	3.243	1.144	0.01	0.325	5.633	99.6	71.0	74.4	73.4	48.5	40.0	-0.69
XHY43	64.687	16.477	4.736	0.789	1.463	2.074	2.864	0.791	0.02	0.243	5.612	99.8	69.1	71.2	71.6	48.8	43.3	-0.67
XHY44	55.026	15.887	14.052	0.949	1.56	1.315	2.981	1.041	0.16	0.394	6.833	100.2	72.0	75.6	74.6	42.9	34.7	-0.73
XHY45	59.873	19.791	4.916	0.699	1.674	1.338	3.908	0.811	0.035	0.207	6.191	99.4	73.3	77.9	75.4	51.3	38.9	-0.70
XHY46	60.447	16.977	6.412	1.127	1.766	1.678	2.948	0.833	0.017	0.34	6.875	99.4	70.2	72.5	74.0	47.2	41.2	-0.68
XHY47	61.249	19.012	4.325	0.433	1.483	1.445	3.883	1.122	0.011	0.077	6.732	99.8	72.5	77.4	74.3	51.3	38.4	-0.70

XHY48	56.205	17.278	10.492	0.572	2.284	1.535	2.888	0.97	0.074	0.059	7.655	100.0	72.5	74.8	75.3	46.3	40.6	-0.69
XHY49	61.941	17.776	5.146	0.944	1.677	2.219	3.189	1.172	0.02	0.271	5.45	99.8	68.5	70.8	71.4	53.7	47.1	-0.64
XHY50	60.913	18.501	5.316	1.021	1.765	2.615	3.096	1.201	0.011	0.455	5.022	99.9	68.7	70.3	70.7	56.4	51.6	-0.60
XHY51	58.931	18.699	6.559	0.672	1.946	2.493	3.13	1.219	0.016	0.248	5.686	99.6	69.7	71.5	71.4	55.9	50.6	-0.61
XHY52	59.806	17.869	6.631	0.726	2.01	2.063	3.117	1.081	0.032	0.28	6.222	99.8	70.6	73.0	72.5	52.0	45.5	-0.65
XHY53	58.897	17.615	6.966	0.769	2.088	1.723	3.205	0.996	0.024	0.245	7.513	100.0	71.2	74.2	73.6	50.1	42.2	-0.67
XHY54	61.1	18.822	4.676	0.591	1.693	2.034	3.671	1.16	0.014	0.172	6	99.9	70.2	73.8	72.0	55.6	45.2	-0.65
XHY55	58.596	18.075	7.04	0.368	2.221	1.222	3.479	1.095	0.027	0.048	7.533	99.7	74.0	78.4	75.8	47.8	37.0	-0.71
XHY56	61.062	16.039	7.632	0.423	2.133	1.68	2.942	0.929	0.031	0.091	6.733	99.7	71.1	74.2	72.9	47.2	39.8	-0.69
XHY57	61.803	17.433	6.633	0.716	1.744	1.672	3.413	1.142	0.037	0.306	4.822	99.7	71.3	75.2	73.0	50.1	40.0	-0.69
BP01	62.941	13.679	4.148	2.045	2.153	2.172	3.094	0.719	0.149	0.179	4.69	100.0	57.2	59.6	66.4	56.9	49.4	-0.62
BP02	60.913	16.072	5.822	2.315	2.001	2.909	3.513	0.712	0.039	0.174	5.63	100.1	56.5	58.4	65.1	67.6	60.2	-0.54
BP03	58.831	16.623	5	2.315	1.845	2.995	3.479	0.729	0.051	0.322	7.69	99.9	57.8	59.6	65.6	67.2	60.4	-0.53
BP04	62.605	16.128	5.841	2.194	2.209	3.917	2.873	0.727	0.053	0.217	3.25	100.0	55.3	55.5	62.8	71.6	70.9	-0.45
BP05	65.205	14.323	6.412	1.946	2.129	2.171	3.206	0.73	0.043	0.161	3.56	99.9	58.4	60.9	67.0	57.6	49.6	-0.62
BP06	66.024	12.608	5.365	2.215	2.132	2.464	3.094	0.851	0.043	0.319	4.87	100.0	54.1	56.7	63.0	59.5	51.4	-0.60
BP07	64.226	12.769	3.151	2.686	1.945	2.615	3.513	1.078	0.055	0.174	7.76	100.0	53.0	56.1	61.1	63.9	53.5	-0.59
BP08	62.064	17.349	5.606	2.573	2.053	1.956	3.572	0.624	0.048	0.188	3.95	100.0	63.6	66.3	71.0	58.1	49.4	-0.62
BP09	65.586	14.323	4.969	1.786	2.07	1.951	3.094	0.684	0.053	0.175	5.32	100.0	57.4	59.7	68.6	55.8	48.0	-0.63
BP10	64.2	17.549	4.303	2.688	1.096	2.477	3.665	0.991	0.013	0.228	2.952	100.2	59.1	61.2	68.5	62.8	55.0	-0.58
BP11	63.876	17.477	4.269	2.681	1.096	2.478	3.647	0.978	0.014	0.226	3.03	99.8	59.1	61.1	68.5	62.6	55.0	-0.58
BP12	67.987	16.526	3.212	2.435	0.65	3.098	2.572	0.463	0.05	0.197	2.415	99.6	58.2	57.9	67.7	57.8	59.3	-0.54
BP13	57.391	21.741	4.81	1.708	1.225	3.314	3.671	1.078	0.048	0.305	4.487	99.8	64.8	65.9	69.7	68.5	64.3	-0.50
BP14	64.808	18.865	2.997	1.78	1.55	1.904	3.443	0.627	0.015	0.157	4.064	100.2	66.0	68.0	73.3	55.2	48.6	-0.63
BP15	61.751	19.534	3.69	1.472	2.125	0.933	4.664	0.574	0.01	0.102	4.962	99.8	70.6	77.4	74.8	56.3	37.3	-0.71

BP16	58.528	22.262	2.785	1.383	2.153	0.642	5.094	0.398	0.009	0.084	6.1	99.4	74.5	81.8	77.2	56.7	35.6	-0.73
BP17	68.052	16.536	2.202	1.303	1.182	1.573	5.079	0.488	0.009	0.13	3.296	99.9	61.9	69.9	67.1	63.9	40.0	-0.69
BP18	59.167	19.724	5.752	1.913	2.018	1.542	4.121	0.786	0.028	0.19	5.014	100.3	67.4	71.2	73.8	58.4	46.0	-0.65
BP19	62.87	19.921	3.199	1.87	1.978	1.163	3.128	0.643	0.007	0.132	5.171	100.1	73.4	75.1	79.0	45.5	40.6	-0.69
BP20	57.375	20.1	7.098	1.805	1.863	1.436	3.507	0.834	0.016	0.173	5.56	99.8	70.2	72.5	76.5	51.6	44.4	-0.66
BP21	60.915	19.862	4.628	2.143	1.842	1.661	2.844	0.783	0.014	0.192	5.122	100.0	69.9	70.2	77.3	48.5	47.4	-0.63
BP22	59.069	21.235	3.911	1.961	2.178	1.125	2.929	0.681	0.007	0.124	6.086	99.3	75.5	76.3	80.9	43.9	41.8	-0.68
BP23	58.063	21.992	3.519	1.768	2.078	0.741	3.578	0.902	0.005	0.14	6.472	99.3	77.7	80.6	81.2	44.8	36.6	-0.72
BP24	62.983	17.741	5.967	1.577	1.823	0.803	2.731	0.76	0.014	0.107	5.717	100.2	76.0	78.0	80.5	37.6	32.9	-0.75
BP25	61.76	20.781	3.35	1.801	1.853	0.672	2.203	0.809	0.006	0.127	6.55	99.9	81.9	81.0	85.6	31.6	33.9	-0.74
BP26	63.915	17.47	6.13	1.814	1.756	0.937	1.941	0.594	0.014	0.112	5.586	100.3	77.1	76.1	82.7	32.2	34.4	-0.73
BP27	67.931	14.376	6.131	1.172	1.119	0.639	3.547	0.609	0.127	0.231	4.541	100.4	70.7	78.1	74.6	40.7	25.5	-0.80
BP28	63.219	20.201	2.131	1.361	1.242	0.75	4.546	0.944	0.004	0.09	5.361	99.8	73.2	79.8	76.6	50.8	32.8	-0.75
BP29	62.16	19.763	3.82	1.392	1.642	0.927	3.719	0.784	0.01	0.037	5.508	99.8	73.6	77.6	78.0	46.9	36.1	-0.72
BP30	58.974	21.561	4.255	2.051	2.363	1.274	2.839	0.557	0.014	0.103	5.939	99.9	74.8	75.0	80.6	45.4	44.7	-0.65
BP31	68.011	16.565	3.142	1.534	0.924	1.443	3.654	0.58	0.013	0.187	3.962	100.0	65.6	69.7	72.3	50.3	38.6	-0.70
BP32	61.616	21.225	2.879	1.78	1.452	1.379	4.002	0.673	0.01	0.183	5.018	100.2	70.5	73.8	76.2	54.0	43.4	-0.66
BP33	65.35	18.768	2.575	1.829	1.23	1.582	3.466	0.617	0.007	0.154	4.279	99.9	67.7	70.1	74.7	51.1	43.5	-0.66
BP34	63.636	20.158	2.545	1.781	1.396	1.402	3.651	0.616	0.007	0.162	4.894	100.2	70.2	72.9	76.3	51.1	42.6	-0.67
BP35	63.834	19.214	3.222	1.428	1.449	0.928	4.683	0.643	0.007	0.149	4.821	100.4	70.3	77.3	74.4	54.6	35.0	-0.73
BP36	62.013	19.375	4.431	1.737	1.88	1.075	3.798	0.605	0.009	0.155	4.978	100.1	71.7	75.7	76.7	49.9	38.5	-0.70
BP37	59.85	22.047	3.471	1.23	1.412	0.788	5.357	0.65	0.008	0.121	5.233	100.2	72.4	80.2	75.6	58.6	35.5	-0.73
BP38	66.663	16.928	3.282	1.211	1.207	1.083	4.185	0.616	0.012	0.134	4.265	99.6	67.6	74.0	72.8	51.4	34.5	-0.73
BP39	64.933	19.485	2.119	1.301	1.154	1.088	4.734	0.687	0.006	0.142	4.555	100.2	69.1	75.7	73.8	56.0	36.8	-0.72
BP40	63.037	18.986	3.726	1.634	1.524	1.334	3.984	0.645	0.015	0.167	4.77	99.8	68.5	72.8	74.4	53.5	40.9	-0.68

BP41	70.518	14.475	2.985	0.768	0.735	0.889	6.01	0.452	0.008	0.108	2.827	99.8	61.3	75.9	64.5	63.0	27.4	-0.79
BP42	61.63	20.356	3.157	1.598	1.755	1.028	4.257	0.622	0.015	0.142	5.223	99.8	71.8	76.8	76.3	52.9	38.4	-0.70
BP43	62.92	19.099	3.713	1.537	1.285	1.354	4.822	0.725	0.013	0.207	4.488	100.2	66.3	72.6	71.9	60.2	40.7	-0.69
BP44	67.466	16.838	3.068	1.348	1.216	1.084	4.054	0.621	0.009	0.157	4.144	100.0	67.9	73.9	73.1	50.4	34.5	-0.73
BP45	61.841	18.063	6.228	1.818	1.68	1.842	3.106	0.858	0.024	0.237	4.721	100.4	66.4	67.9	73.8	51.9	47.2	-0.64

Table DR3.

Trace element contents (%) of the analyzed mudstones from Xiahuayuan and Beipiao successions.

Sample	XHY01	XHY02	XHY03	XHY04	XHY05	XHY06	XHY07	XHY08	XHY09	XHY10	XHY11	XHY12	XHY13	XHY14	XHY15	XHY16	XHY17	XHY18	XHY19	XHY20	XHY21	XHY22
Li	38.4	32.1	35.4	26.5	35.5	46.3	34.3	36.1	62.6	25.1	34.5	29.0	29.0	24.9	26.9	32.8	28.9	39.0	25.4	32.5	33.6	49.2
Be	3.93	3.79	3.94	4.12	3.91	5.15	4.61	3.57	3.37	1.77	2.35	2.01	1.86	1.69	1.95	2.28	2.19	1.70	2.35	4.02	3.57	3.98
Sc	17.6	18.8	13.4	13.0	17.3	15.8	17.6	14.9	17.0	7.87	11.6	9.85	9.94	8.98	10.1	11.6	9.45	8.89	10.0	16.1	15.0	16.1
V	135	129	98.1	96.6	131	107	132	113	138	52.8	77.3	62.7	66.8	58.5	67.7	75.7	62.3	82.8	79.1	130	109	122
Cr	80.1	82.6	71.0	58.7	73.9	58.6	68.2	67.8	87.3	36.4	58.0	46.9	50.9	47.0	52.5	57.7	50.2	28.5	37.0	73.3	62.6	77.2
Co	12.6	12.7	21.5	12.2	8.92	21.5	14.3	14.8	13.8	7.87	12.3	9.10	9.04	8.12	9.37	12.0	8.65	9.47	9.31	11.8	14.2	14.2
Ni	41.8	43.9	43.4	26.5	35.3	35.1	38.8	46.6	45.5	16.8	27.9	19.4	21.2	18.8	22.5	29.5	22.6	10.7	14.6	40.2	37.9	48.1
Cu	41.5	40.0	27.5	24.6	38.8	31.0	38.6	37.0	47.8	14.7	23.9	17.0	17.1	14.9	16.7	23.4	16.5	10.5	15.9	29.3	31.6	37.6
Zn	111	111	126	108	96.6	135	85.2	98.0	169	46.4	79.9	53.1	54.6	49.4	59.9	74.5	58.8	65.3	56.7	86.7	72.1	98.9
Ga	25.5	24.8	23.4	24.9	27.5	27.2	25.9	24.7	25.2	15.0	19.0	17.1	15.2	15.3	16.4	18.6	15.3	17.5	22.6	25.2	23.2	24.4
Rb	130	103	103	111	135	147	123	109	103	90.0	136	103	104	101	108	157	112	52.3	104	126	103	108
Sr	181	194	217	230	158	151	154	178	204	255	179	257	211	233	221	171	181	602	524	149	206	226
Y	41.8	49.2	43.0	46.7	44.6	44.1	41.3	42.7	33.6	19.2	28.4	24.0	26.3	23.9	24.1	24.8	22.8	14.3	21.1	35.2	42.5	46.5
Zr	274	283	288	306	267	239	277	271	201	164	212	293	291	245	263	219	220	186	295	262	275	285
Nb	28.1	25.3	27.8	31.2	30.8	31.6	29.4	26.2	19.4	11.8	14.2	15.2	12.9	12.6	13.4	13.4	11.9	11.7	15.0	27.2	28.1	27.2
Sn	3.41	2.62	2.91	3.35	3.55	3.28	3.46	3.18	2.50	1.52	2.19	1.85	1.91	1.71	1.93	2.31	1.93	1.15	1.54	3.41	3.24	3.18
Cs	6.25	2.82	4.11	3.46	5.96	16.3	5.12	5.37	10.9	3.91	7.82	5.20	5.21	5.12	5.46	9.19	5.65	1.14	2.49	6.22	4.33	4.72
Ba	575	537	579	638	438	514	491	565	527	410	389	431	819	737	761	1177	434	760	1140	486	822	704
La	61.0	61.7	58.1	55.0	71.3	80.1	71.0	58.0	58.6	30.9	41.4	37.2	33.8	36.0	34.8	37.8	31.5	34.0	41.1	63.6	58.1	68.0
Ce	131	130	122	115	146	168	146	121	128	61.0	87.5	76.1	65.8	71.7	68.5	76.7	60.5	66.1	79.0	134	121	144
Pr	14.3	15.0	14.0	13.0	16.2	18.0	16.1	13.4	14.7	7.04	10.0	8.43	7.73	8.18	7.84	8.61	7.15	7.44	8.72	14.3	13.8	16.6
Nd	53.4	58.8	52.7	50.3	59.1	64.2	58.8	51.5	58.1	26.5	38.3	31.2	29.7	30.9	29.3	32.2	27.5	28.1	32.8	52.4	51.0	61.8

Sm	9.28	10.4	9.24	8.93	9.20	10.6	8.95	8.66	9.95	4.78	6.94	5.71	5.70	5.64	5.27	5.78	5.01	4.46	5.74	7.41	9.06	10.8
Eu	1.81	2.69	1.87	1.92	1.70	1.66	1.59	1.69	2.06	1.13	1.42	1.23	1.23	1.26	1.18	1.32	1.07	1.23	1.52	1.27	1.85	2.37
Gd	7.84	10.1	8.43	8.78	7.71	8.36	6.93	7.39	8.30	4.01	5.92	4.89	4.81	4.79	4.58	5.18	4.49	3.57	4.83	5.44	8.00	9.83
Tb	1.22	1.54	1.28	1.30	1.17	1.30	1.11	1.12	1.19	0.59	0.87	0.70	0.75	0.74	0.69	0.74	0.68	0.47	0.68	0.92	1.23	1.44
Dy	7.46	8.63	7.87	7.79	7.45	7.65	6.95	6.91	6.32	3.55	5.17	4.18	4.58	4.33	4.11	4.40	3.98	2.64	4.06	5.98	7.50	8.44
Ho	1.41	1.57	1.42	1.50	1.50	1.48	1.43	1.37	1.13	0.67	1.03	0.81	0.91	0.83	0.81	0.87	0.78	0.52	0.73	1.20	1.47	1.57
Er	4.11	4.53	4.07	4.33	4.45	4.30	4.17	4.14	3.24	1.88	2.85	2.30	2.60	2.41	2.41	2.45	2.21	1.42	1.97	3.65	4.20	4.44
Tm	0.62	0.67	0.61	0.64	0.67	0.66	0.65	0.60	0.45	0.27	0.44	0.34	0.39	0.36	0.38	0.39	0.35	0.21	0.30	0.56	0.66	0.67
Yb	4.21	4.30	4.01	4.25	4.67	4.64	4.46	4.03	2.97	1.85	2.88	2.30	2.61	2.37	2.43	2.52	2.30	1.38	1.96	3.80	4.25	4.28
Lu	0.63	0.64	0.59	0.62	0.69	0.67	0.66	0.61	0.46	0.27	0.44	0.33	0.39	0.37	0.37	0.37	0.34	0.21	0.31	0.58	0.62	0.63
Hf	7.32	6.97	7.45	8.00	7.42	6.78	7.59	7.24	5.27	4.32	5.79	7.68	7.59	6.49	6.75	5.82	5.98	4.79	7.73	6.92	7.11	7.09
Ta	1.65	1.47	1.66	1.90	1.90	1.94	1.82	1.60	1.12	0.86	1.01	1.07	0.94	0.92	0.93	0.95	0.83	0.82	1.13	1.61	1.62	1.51
Tl	1.02	0.77	0.85	0.94	1.12	1.51	0.85	0.88	0.70	0.57	0.82	0.66	0.62	0.62	0.65	0.87	0.67	0.34	0.58	0.86	0.66	0.70
Pb	35.8	22.4	25.5	30.9	29.7	60.6	23.6	25.7	27.6	13.5	23.6	16.9	15.3	13.9	18.2	21.5	14.9	13.3	13.4	23.8	23.3	19.9
Th	10.8	8.20	8.45	9.95	12.9	14.3	12.2	9.90	8.09	8.37	11.9	11.2	10.3	9.96	11.0	12.4	9.76	6.86	10.5	11.6	9.75	9.15
U	2.76	2.14	2.22	2.59	3.00	3.60	2.76	2.53	2.59	1.91	2.40	2.36	2.51	2.18	2.35	2.17	2.70	1.60	2.16	2.79	4.01	5.89

Sample	XHY23	XHY24	XHY25	XHY26	XHY27	XHY28	XHY29	XHY30	XHY31	XHY32	XHY33	XHY34	XHY35	XHY36	XHY37	XHY38	XHY39	XHY40	XHY41	XHY42	XHY43	XHY44
Li	23.9	34.7	35.5	42.8	42.6	31.5	36.3	39.1	34.4	32.7	36.8	65.7	43.4	44.6	46.0	64.7	63.1	63.8	24.3	34.3	37.1	27.6
Be	4.23	4.58	5.94	4.43	4.14	4.13	4.23	4.29	4.66	5.27	4.05	4.07	3.48	3.82	3.61	3.97	3.53	3.37	4.53	3.52	3.38	5.09
Sc	10.7	15.6	20.5	15.4	15.5	11.6	12.7	17.9	20.3	22.8	16.4	20.6	16.1	16.0	9.66	15.9	14.8	12.1	14.2	17.0	11.6	15.2
V	90.9	123	139	123	107	94.0	101	128	141	152	129	154	112	121	79.8	129	122	88.4	101	133	79.3	114
Cr	50.3	82.8	74.8	77.3	71.2	66.9	58.2	73.5	78.7	84.1	81.5	92.4	77.1	94.1	48.0	68.1	66.5	52.4	74.1	76.5	38.3	72.1
Co	24.7	17.8	16.0	12.4	16.9	14.8	9.96	14.0	14.6	9.68	13.0	22.3	14.8	15.3	19.4	14.6	13.0	16.0	17.5	6.78	12.3	16.1
Ni	38.3	32.2	36.9	37.3	50.2	33.0	31.0	47.1	44.2	36.3	40.9	62.2	47.6	47.1	34.5	50.3	39.2	40.0	39.1	38.4	32.1	34.6

Cu	24.4	31.5	44.9	41.3	36.2	26.7	27.3	39.8	52.7	42.3	36.1	56.1	36.1	35.3	20.9	36.0	29.1	28.1	27.6	36.3	25.3	36.2
Zn	53.7	93.6	90.8	134	108	101	91.5	115	127	86.7	139	137	122	107	77.9	155	133	117	112	124	84.4	77.3
Ga	17.0	26.3	27.8	26.1	24.3	22.6	24.4	25.8	25.4	28.7	24.3	27.9	24.4	25.4	22.9	28.6	27.7	21.5	23.9	25.3	26.5	22.8
Rb	84.2	114	134	110	105	92.7	92.9	120	117	142	109	142	89.3	98.0	88.7	165	166	105	104	108	135	107
Sr	142	220	259	220	226	232	203	174	188	158	199	136	244	234	198	120	119	175	193	160	110	150
Y	38.2	46.8	79.3	46.8	45.1	36.1	50.3	42.6	46.8	57.2	42.6	42.9	46.7	36.8	32.4	45.0	40.1	35.7	46.6	45.5	38.0	49.2
Zr	182	305	351	279	266	249	309	286	287	375	267	219	228	252	258	243	257	201	286	282	266	248
Nb	17.7	30.2	32.3	29.5	27.0	24.2	31.5	29.3	27.3	35.7	24.6	24.8	23.1	23.6	25.6	23.6	27.9	20.7	26.7	27.8	30.9	23.9
Sn	2.38	3.59	4.24	3.53	3.14	2.54	3.33	3.42	3.20	3.58	2.88	3.03	2.55	2.59	2.69	3.29	3.60	2.23	2.86	3.31	3.78	2.87
Cs	4.60	4.14	6.38	4.86	4.45	2.88	2.82	6.03	5.00	5.05	4.65	16.1	2.42	2.61	4.18	16.0	18.8	8.19	4.22	3.76	11.9	5.10
Ba	335	548	550	604	553	571	474	455	430	555	549	424	590	660	604	362	403	693	541	465	311	444
La	48.9	64.5	88.3	59.9	57.0	45.0	54.1	68.1	68.1	70.8	57.9	65.6	49.9	49.9	49.0	80.2	62.7	62.5	59.3	59.5	62.9	57.1
Ce	94.6	132	193	127	123	99.0	114	147	144	152	123	136	114	106	104	167	145	136	128	126	131	115
Pr	10.1	14.8	23.1	14.0	14.0	11.3	13.3	16.8	16.1	16.5	14.3	15.1	13.4	12.4	10.8	19.1	15.3	15.1	15.1	14.5	15.3	13.1
Nd	36.9	54.1	93.2	51.6	52.4	44.0	51.1	64.0	60.9	61.7	56.1	56.1	54.7	49.7	40.7	71.5	57.9	58.9	60.4	56.0	56.7	51.4
Sm	5.96	9.13	17.2	9.08	9.23	7.66	9.25	10.9	10.3	9.95	9.67	9.71	11.1	8.74	6.76	12.2	9.91	10.2	11.6	9.88	9.40	9.20
Eu	1.30	1.90	3.60	1.59	2.00	1.60	1.98	2.15	1.88	1.95	2.05	1.90	2.61	2.01	1.28	2.38	1.89	1.98	2.86	2.04	1.55	2.17
Gd	5.79	8.30	17.2	8.11	8.81	7.05	8.82	9.54	8.74	9.18	8.52	8.23	10.6	7.65	5.36	10.2	8.17	8.74	10.6	8.64	7.38	8.27
Tb	0.93	1.26	2.49	1.24	1.33	1.03	1.38	1.38	1.29	1.46	1.28	1.23	1.54	1.15	0.88	1.50	1.21	1.23	1.48	1.30	1.14	1.30
Dy	5.67	7.76	13.8	7.61	8.18	6.40	8.36	8.08	8.36	9.50	7.56	7.47	8.66	6.52	5.29	8.23	6.94	6.84	8.95	7.91	6.82	8.21
Ho	1.10	1.51	2.47	1.53	1.51	1.20	1.59	1.47	1.56	1.79	1.40	1.45	1.56	1.21	1.07	1.49	1.31	1.22	1.56	1.51	1.28	1.58
Er	3.31	4.49	6.51	4.45	4.33	3.44	4.57	4.27	4.68	5.26	4.10	4.19	4.22	3.48	3.24	4.30	3.97	3.42	4.41	4.34	4.05	4.62
Tm	0.49	0.68	0.92	0.69	0.65	0.52	0.70	0.65	0.71	0.77	0.61	0.63	0.60	0.51	0.50	0.64	0.60	0.50	0.64	0.63	0.62	0.66
Yb	3.39	4.66	5.90	4.60	4.30	3.42	4.64	4.35	4.82	5.06	4.19	4.36	4.03	3.47	3.49	4.35	4.17	3.34	4.37	4.39	4.41	4.50
Lu	0.51	0.68	0.85	0.67	0.64	0.50	0.68	0.66	0.72	0.74	0.62	0.65	0.59	0.49	0.51	0.65	0.64	0.48	0.65	0.65	0.67	0.69

Hf	4.68	7.80	9.03	7.24	7.13	6.37	8.04	7.64	7.61	9.09	6.81	6.06	5.89	6.03	6.89	6.37	6.84	5.31	7.23	7.14	7.55	6.56
Ta	1.09	1.79	1.99	1.72	1.66	1.38	1.79	1.76	1.71	2.03	1.39	1.45	1.38	1.32	1.48	1.35	1.60	1.22	1.60	1.68	1.84	1.40
Tl	0.56	0.75	0.94	0.78	0.78	0.71	0.68	0.91	0.83	1.07	0.81	1.13	0.61	0.66	0.78	1.94	1.66	0.98	0.74	0.73	1.30	0.78
Pb	13.6	25.4	23.7	26.9	24.2	20.9	17.9	31.6	25.1	19.3	26.7	50.2	29.8	20.5	28.3	51.5	42.0	32.9	31.2	22.5	32.2	23.0
Th	7.55	10.7	13.5	11.3	9.32	7.38	9.79	10.9	11.1	12.6	8.66	12.1	7.27	7.32	8.90	11.3	12.0	7.79	8.87	10.3	11.5	9.25
U	13.3	4.31	3.66	3.05	2.37	2.16	2.50	2.93	2.37	2.83	2.38	3.11	2.11	2.11	2.27	2.97	2.96	2.07	2.43	2.51	2.79	2.40

Sample	XHY45	XHY46	XHY47	XHY48	XHY49	XHY50	XHY51	XHY52	XHY53	XHY54	XHY55	XHY56	XHY57	BP01	BP02	BP03	BP04	BP05	BP06	BP07	BP08	BP09
Li	36.1	52.4	30.5	59.7	36.5	33.0	39.1	42.4	48.1	33.1	49.5	57.0	34.7	37.4	66.4	24.9	15.1	14.9	22.4	13.1	8.24	8.27
Be	4.50	3.69	4.50	3.67	3.29	4.06	4.41	4.69	4.11	4.22	4.38	3.76	4.42	4.35	3.79	4.34	1.35	2.39	2.42	3.20	3.71	3.66
Sc	14.3	13.8	20.0	18.3	14.6	15.3	16.1	16.7	19.2	16.3	21.0	16.8	16.3	12.9	21.0	14.1	14.6	10.8	8.26	9.92	8.23	8.08
V	80.3	87.1	133	146	123	111	120	128	138	120	170	135	125	98.2	157	102	108	85.8	62.7	76.9	32.4	31.9
Cr	39.3	53.1	72.5	87.4	85.8	75.3	87.7	82.6	70.6	70.9	96.8	80.1	82.7	56.7	92.9	73.2	20.8	53.9	41.9	35.8	18.9	18.8
Co	11.1	13.4	7.54	25.8	15.6	10.6	20.6	21.2	18.4	14.4	23.6	22.0	15.0	9.67	22.7	17.6	8.32	5.12	5.33	2.17	3.14	3.15
Ni	24.1	30.7	22.9	61.9	39.6	33.9	48.4	50.8	38.9	30.7	52.3	56.4	38.5	31.0	63.3	39.2	5.68	13.4	10.02	7.73	8.08	7.86
Cu	28.2	33.1	37.4	34.9	33.5	29.2	38.7	38.5	39.2	26.3	43.8	42.6	33.4	27.2	56.9	27.9	16.1	27.3	16.6	18.8	8.04	8.12
Zn	94.7	112	104	113	89.2	92.3	106	118	107	86.1	96.5	105	117	91.4	141	112	78.5	80.0	56.5	47.3	72.7	72.7
Ga	29.8	25.2	28.7	27.9	25.3	25.5	26.5	25.5	26.6	26.8	27.8	23.9	24.7	24.4	28.7	24.3	19.2	24.4	22.4	22.4	27.4	26.9
Rb	143	121	152	127	102	99.7	102	107	132	128	144	116	111	92.4	143	103	163	113	106	156	181	180
Sr	129	189	130	138	218	195	195	183	235	224	202	206	233	202	139	198	410	279	201	139	122	122
Y	60.6	51.5	45.6	34.9	40.2	51.1	49.6	47.5	52.2	50.5	38.4	31.0	47.4	49.2	43.6	47.1	20.0	26.0	22.6	28.6	32.1	32.0
Zr	306	232	290	203	290	354	328	292	249	380	277	204	341	305	223	287	154	274	243	271	317	310
Nb	37.4	26.3	29.6	24.1	27.6	32.6	31.8	29.5	27.1	35.6	28.1	19.7	27.3	30.8	25.2	26.9	7.29	19.7	13.8	20.2	19.7	19.7
Sn	3.95	3.33	3.53	2.92	2.97	3.35	3.50	3.30	3.26	3.91	3.58	2.45	2.98	3.14	3.04	2.66	1.03	2.62	2.29	2.75	2.84	2.84
Cs	14.3	14.8	19.0	13.5	3.92	2.49	3.04	4.47	18.7	7.71	10.2	7.82	4.11	2.73	16.5	4.12	3.50	10.5	1.78	4.66	12.8	12.7

Ba	478	563	496	412	537	524	528	500	506	551	505	513	566	467	428	543	672	575	742	618	501	504
La	140	83.3	101	49.0	65.1	63.3	65.9	65.4	101	74.9	52.2	51.0	64.6	52.8	65.9	60.0	33.6	54.6	49.1	47.8	73.6	73.2
Ce	273	173	218	129	135	132	140	142	204	148	108	109	133	112	138	129	65.2	110	90.8	84.3	143	143
Pr	31.2	19.2	24.3	12.5	15.5	15.0	15.5	15.2	21.7	16.0	11.0	11.9	15.0	13.3	15.5	15.4	8.08	13.0	10.6	9.55	16.1	16.1
Nd	115	73.0	95.1	48.6	59.8	59.2	60.8	59.0	80.9	58.2	40.0	47.0	58.5	52.1	57.3	60.4	30.9	49.9	37.8	34.8	57.6	58.8
Sm	18.2	12.6	15.5	8.87	10.3	10.2	10.1	10.1	12.8	8.35	5.99	8.05	9.78	9.59	9.81	11.6	5.24	8.47	6.56	6.02	9.47	9.79
Eu	2.46	2.22	2.46	1.66	1.95	2.22	2.03	1.92	2.31	1.43	1.09	1.76	2.13	1.98	1.87	2.88	1.59	1.59	1.29	1.08	1.51	1.50
Gd	13.8	11.2	11.1	7.14	8.05	8.66	8.19	8.31	9.96	6.38	4.52	6.39	8.34	8.52	8.19	10.4	4.41	6.46	5.23	5.20	7.62	7.66
Tb	1.93	1.64	1.53	1.01	1.20	1.36	1.38	1.26	1.50	1.10	0.84	0.95	1.34	1.31	1.19	1.50	0.60	0.85	0.71	0.80	1.07	1.08
Dy	11.4	9.48	8.85	6.04	7.27	8.46	8.52	7.91	9.10	7.86	6.26	5.68	8.49	8.35	7.81	9.12	3.53	5.03	4.13	4.73	5.96	5.93
Ho	2.09	1.77	1.63	1.12	1.40	1.70	1.70	1.61	1.80	1.66	1.30	1.10	1.65	1.56	1.44	1.53	0.63	0.85	0.77	0.91	1.12	1.09
Er	6.11	4.72	4.77	3.38	4.10	4.89	4.85	4.58	5.18	5.06	3.93	3.12	4.70	4.65	4.30	4.45	1.85	2.52	2.23	2.65	2.99	3.04
Tm	0.86	0.67	0.68	0.51	0.60	0.69	0.69	0.63	0.71	0.74	0.57	0.45	0.65	0.66	0.61	0.64	0.26	0.33	0.32	0.41	0.46	0.46
Yb	5.95	4.46	4.75	3.44	4.11	4.80	4.85	4.42	5.24	5.27	4.07	3.30	4.86	4.55	4.34	4.40	1.73	2.34	2.02	2.72	3.05	3.13
Lu	0.89	0.67	0.71	0.53	0.62	0.73	0.72	0.66	0.79	0.79	0.61	0.49	0.73	0.66	0.64	0.64	0.25	0.35	0.31	0.41	0.45	0.46
Hf	8.89	6.45	7.55	5.51	7.27	8.72	8.20	7.31	6.69	9.67	7.16	5.23	8.19	7.85	6.04	7.48	3.81	7.15	6.35	7.01	8.66	8.36
Ta	2.18	1.62	1.74	1.34	1.69	2.02	1.94	1.77	1.62	2.17	1.71	1.17	1.67	1.81	1.47	1.59	0.34	1.41	1.17	1.46	1.51	1.53
Tl	1.17	1.08	1.27	0.82	0.71	0.65	0.65	0.72	0.91	0.85	0.92	0.73	0.71	0.59	1.13	0.70	0.39	0.62	0.59	0.71	0.65	0.69
Pb	19.8	26.4	31.8	30.1	22.3	25.8	28.1	26.7	27.2	29.6	29.5	23.2	20.1	17.9	49.5	30.5	11.3	19.2	18.1	13.6	18.8	18.6
Th	16.0	10.9	12.3	10.8	9.57	10.2	10.9	10.7	12.0	12.0	13.2	8.98	9.66	9.44	12.2	8.91	2.62	16.5	13.2	15.7	12.1	12.1
U	3.53	2.77	3.29	2.73	2.13	2.40	2.41	2.47	3.08	2.81	2.68	2.26	2.09	2.45	3.17	2.39	0.66	5.43	3.43	4.00	3.08	3.25

Sample	BP10	BP11	BP12	BP13	BP14	BP15	BP16	BP17	BP18	BP19	BP20	BP21	BP22	BP23	BP24	BP25	BP26	BP27	BP28	BP29	BP30	BP31
Li	45.6	14.8	22.4	25.1	11.1	9.94	37.4	9.54	10.6	12.0	12.5	14.5	11.9	8.79	11.6	15.3	15.3	19.3	10.7	15.1	14.3	21.9
Be	2.27	1.43	1.37	2.05	2.30	3.13	2.04	3.03	3.13	3.16	3.17	2.43	2.66	2.95	3.25	2.18	2.69	3.49	3.65	3.73	4.16	2.59

Sc	11.9	14.8	6.12	13.8	9.24	10.6	8.93	7.42	12.4	10.3	12.1	10.6	9.50	13.1	10.1	10.4	7.70	10.2	10.9	13.0	9.90	8.21
V	104	108	43.5	122	54.0	68.2	73.7	37.9	95.3	63.2	91.9	85.2	58.2	73.6	84.5	74.3	70.7	96.7	83.8	94.4	64.4	62.3
Cr	66.6	20.9	3.64	8.62	45.7	67.3	33.1	23.9	86.8	52.2	60.4	54.2	41.0	31.4	37.7	35.6	40.8	44.3	34.0	65.9	78.7	40.7
Co	11.9	8.43	5.84	6.98	3.50	5.16	9.85	3.02	10.4	2.92	6.28	5.09	2.27	1.32	7.58	1.88	7.06	11.8	1.07	7.77	12.2	5.24
Ni	29.1	5.39	3.00	5.73	9.49	16.2	12.9	7.44	24.5	12.7	18.3	12.9	7.20	5.04	13.0	8.27	14.4	19.3	5.24	21.9	28.2	10.27
Cu	37.3	16.2	10.6	28.6	12.3	29.2	12.5	9.20	37.9	20.0	40.0	27.1	16.4	17.6	27.6	16.4	20.7	37.2	13.1	30.0	22.0	16.6
Zn	112	77.1	51.3	76.6	102	91.3	59.1	51.0	134	81.3	93.1	79.0	52.3	33.9	72.9	50.4	105	79.0	27.2	75.0	115	56.3
Ga	16.0	19.3	18.9	26.9	22.9	25.9	20.2	20.7	25.6	26.7	25.0	24.3	26.8	28.5	23.5	24.9	21.9	18.8	23.6	26.0	29.1	22.4
Rb	87.7	164	94.0	148	112	158	65.6	139	146	138	124	112	133	150	122	85.0	93.9	122	132	184	132	106
Sr	101	413	531	434	228	171	629	181	267	186	246	277	205	185	182	152	172	117	123	164	210	200
Y	27.5	20.0	11.8	23.8	32.5	32.7	17.6	29.6	27.6	37.8	28.7	26.2	31.5	32.1	28.5	26.0	26.4	37.4	21.0	27.8	33.0	22.7
Zr	205	158	156	208	334	278	325	307	255	326	276	280	361	352	258	241	258	199	266	279	274	243
Nb	15.7	7.15	7.56	8.87	20.4	21.2	12.0	18.5	18.2	27.6	18.7	19.1	26.5	31.2	19.7	17.0	18.4	14.4	15.3	22.9	25.1	13.8
Sn	2.27	1.00	0.88	1.17	2.62	2.84	1.31	2.58	2.35	3.85	2.50	2.58	3.62	3.54	2.80	2.48	2.68	1.95	2.28	3.10	3.62	2.26
Cs	5.89	3.52	1.32	1.46	4.64	9.96	1.37	3.43	11.2	10.4	8.56	10.3	12.0	10.2	8.97	7.36	7.73	5.80	2.52	13.1	10.7	1.86
Ba	335	676	793	738	620	530	861	783	627	473	565	572	475	453	428	409	421	716	570	516	475	746
La	34.6	33.7	32.9	56.7	54.8	63.0	46.5	49.5	54.0	57.9	47.1	53.8	64.8	51.0	48.5	57.3	54.4	43.2	24.1	56.5	65.3	49.5
Ce	70.8	65.4	62.9	98.5	102	133	82.3	94.4	104	108	95.9	110	131	102	96.7	100	107	79.7	37.9	114	124	91.2
Pr	8.14	7.98	7.23	11.9	11.7	15.3	8.79	10.6	11.9	12.9	10.8	12.9	15.8	12.2	10.7	12.4	11.7	8.37	4.45	11.2	13.8	10.4
Nd	32.3	31.7	28.9	45.9	44.1	58.3	32.8	38.5	45.4	47.2	41.1	49.8	55.7	45.0	39.0	46.0	42.7	31.4	16.7	38.6	48.6	38.4
Sm	6.01	5.29	4.43	7.05	7.43	9.88	5.35	6.44	7.63	9.17	7.01	8.21	9.72	8.92	7.03	7.59	7.49	6.35	3.51	6.59	8.46	6.64
Eu	1.34	1.55	1.31	1.86	1.56	1.77	1.43	1.30	1.57	1.25	1.42	1.56	1.43	1.48	1.31	1.44	1.42	1.45	0.89	1.22	1.33	1.33
Gd	5.04	4.30	3.28	5.64	6.45	7.83	4.08	5.50	6.17	7.77	5.82	6.17	7.23	6.84	5.96	5.86	5.98	5.89	3.39	5.19	6.94	5.09
Tb	0.78	0.62	0.44	0.77	0.96	1.12	0.60	0.82	0.89	1.16	0.87	0.87	1.01	0.98	0.85	0.81	0.86	0.88	0.53	0.81	1.00	0.70
Dy	4.90	3.58	2.39	4.37	5.78	6.70	3.46	5.05	5.51	6.69	5.42	5.22	5.86	5.66	5.11	4.45	4.65	5.57	3.41	4.92	5.84	4.16

Ho	0.93	0.63	0.43	0.83	1.10	1.19	0.63	1.00	0.96	1.28	0.99	0.89	1.11	1.10	1.02	0.86	0.89	1.14	0.66	0.95	1.13	0.77
Er	2.60	1.90	1.20	2.15	3.02	3.18	1.85	2.78	2.71	3.66	2.75	2.49	3.19	3.08	2.89	2.36	2.60	3.45	1.91	2.70	3.27	2.24
Tm	0.39	0.26	0.16	0.29	0.43	0.44	0.26	0.40	0.36	0.50	0.38	0.34	0.44	0.43	0.42	0.36	0.36	0.53	0.29	0.39	0.46	0.33
Yb	2.56	1.76	1.06	2.02	3.01	3.11	1.94	2.92	2.66	3.51	2.71	2.41	2.86	2.74	2.83	2.36	2.47	3.49	1.75	2.44	2.99	2.11
Lu	0.39	0.26	0.16	0.29	0.45	0.45	0.30	0.44	0.40	0.51	0.42	0.35	0.42	0.40	0.42	0.34	0.37	0.55	0.26	0.36	0.44	0.30
Hf	5.02	3.76	3.74	4.86	8.28	7.14	7.81	7.58	6.51	8.98	7.16	7.21	9.59	8.99	6.73	6.60	6.88	5.08	6.66	7.48	7.88	6.53
Ta	1.07	0.32	0.35	0.43	1.36	1.34	0.90	1.34	1.09	2.02	1.38	1.34	2.05	1.87	1.50	1.17	1.48	0.96	1.08	1.56	1.97	1.16
Tl	0.51	0.41	0.30	0.45	0.94	0.68	0.39	0.55	0.58	0.62	0.59	0.60	0.63	0.70	0.63	0.63	0.90	0.75	0.60	0.99	0.80	0.60
Pb	19.9	11.6	9.76	12.4	16.6	23.5	13.0	18.0	23.8	20.7	23.4	19.0	20.4	15.0	25.1	10.1	28.5	18.2	12.4	15.7	29.5	18.4
Th	10.0	2.72	2.71	3.46	12.4	12.4	8.34	11.8	9.15	24.2	15.8	16.4	26.1	22.8	17.6	12.5	18.0	12.2	11.2	19.1	26.0	13.4
U	2.26	0.65	0.61	1.14	3.46	3.26	1.96	3.07	2.80	5.59	4.13	5.47	6.22	6.60	4.48	3.11	4.12	3.36	3.02	3.90	5.29	3.41

Sample	BP32	BP33	BP34	BP35	BP36	BP37	BP38	BP39	BP40	BP41	BP42	BP43	BP44	BP45
Li	11.7	14.5	13.5	12.6	12.4	10.4	16.5	13.9	12.7	21.0	11.9	15.1	16.4	21.7
Be	3.33	2.49	2.83	3.29	2.95	4.03	2.76	3.25	3.06	2.65	3.24	2.53	2.55	1.93
Sc	9.94	7.61	8.37	9.57	10.4	11.1	8.71	10.5	9.52	7.37	8.99	9.24	8.23	11.7
V	76.0	68.5	61.5	64.8	69.6	67.5	70.9	78.4	78.9	53.7	62.3	74.2	65.8	113
Cr	56.8	50.6	47.2	46.8	59.3	48.9	52.2	35.7	64.3	27.0	45.9	50.6	52.2	95.9
Co	3.89	3.23	2.41	3.04	4.22	5.22	6.35	2.17	4.40	5.44	4.66	4.81	3.10	7.44
Ni	9.77	8.87	8.93	10.6	16.0	12.2	16.6	7.51	14.7	14.3	11.8	14.0	12.8	27.5
Cu	16.3	13.7	12.9	15.6	20.2	19.6	25.5	19.0	21.9	18.8	17.0	20.4	18.4	35.4
Zn	67.4	65.7	51.2	50.0	81.1	64.2	70.5	47.4	70.3	51.2	56.4	65.0	54.6	88.6
Ga	26.6	23.3	24.1	23.6	25.4	27.3	20.5	22.6	23.4	18.3	24.4	23.8	20.4	21.2
Rb	146	118	122	153	155	208	131	156	145	156	168	159	128	108
Sr	218	246	216	131	166	117	149	141	207	112	143	205	155	243

Y	30.9	25.9	29.3	33.0	37.0	40.5	28.5	28.8	30.1	23.9	37.9	30.5	28.3	21.8
Zr	337	302	307	291	337	325	211	268	320	182	332	300	297	212
Nb	23.4	20.2	20.9	23.0	24.5	23.4	17.1	19.7	20.9	13.7	24.9	20.9	18.6	13.9
Sn	3.32	2.92	3.03	3.40	3.61	4.22	2.46	2.77	3.14	2.48	3.72	3.00	2.69	2.01
Cs	5.11	2.89	3.15	6.08	8.58	13.4	5.41	4.71	6.28	3.03	11.9	5.67	3.93	5.78
Ba	652	701	629	603	557	521	598	630	620	627	559	724	605	670
La	58.3	55.9	63.2	59.6	72.7	87.4	51.1	49.0	59.4	47.7	67.3	54.5	56.9	41.3
Ce	104	100	110	103	137	160	92.6	85.8	103	83.8	122	101	99.8	75.8
Pr	12.2	11.8	13.3	12.8	16.4	19.6	10.5	9.78	12.4	9.17	14.2	11.9	11.7	9.42
Nd	43.6	41.8	47.8	46.2	59.3	68.5	38.6	36.1	46.3	34.0	53.3	45.6	44.6	36.1
Sm	7.69	7.30	8.00	8.07	10.6	11.7	6.85	6.26	7.84	5.70	9.07	7.73	7.38	6.40
Eu	1.28	1.25	1.37	1.31	1.61	1.72	1.20	1.13	1.34	1.05	1.48	1.44	1.29	1.41
Gd	6.29	5.72	6.31	6.72	8.15	9.20	5.58	5.22	6.15	4.53	7.84	6.08	5.80	5.06
Tb	0.90	0.82	0.89	0.99	1.17	1.31	0.81	0.81	0.91	0.68	1.10	0.91	0.85	0.70
Dy	5.42	4.74	5.02	5.55	6.61	7.74	4.87	4.85	5.10	4.11	6.64	5.12	4.79	4.01
Ho	1.03	0.91	0.95	1.12	1.24	1.40	0.95	0.91	0.96	0.80	1.26	0.96	0.89	0.75
Er	2.91	2.53	2.78	2.99	3.33	3.67	2.71	2.60	2.85	2.12	3.42	2.68	2.50	1.96
Tm	0.45	0.39	0.41	0.47	0.52	0.56	0.42	0.42	0.44	0.33	0.52	0.43	0.40	0.31
Yb	3.00	2.65	2.86	3.03	3.29	3.58	2.69	2.79	2.95	2.21	3.43	2.73	2.57	1.96
Lu	0.45	0.39	0.42	0.46	0.48	0.52	0.40	0.42	0.43	0.32	0.51	0.41	0.38	0.29
Hf	8.93	7.61	7.86	7.81	8.68	9.57	5.55	7.19	8.11	5.29	8.85	7.76	7.56	5.54
Ta	1.83	1.50	1.58	1.77	1.90	2.15	1.18	1.49	1.49	1.26	1.87	1.49	1.36	0.93
Tl	0.77	0.55	0.60	0.66	0.76	1.37	0.82	0.76	0.62	0.67	0.81	0.94	0.60	0.51
Pb	19.3	21.0	14.5	14.3	22.3	18.4	18.0	13.6	16.4	19.0	19.1	15.4	13.4	11.6
Th	20.8	17.2	18.5	20.8	23.0	27.3	13.2	16.3	17.7	14.8	22.2	17.7	16.1	9.26

U	4.81	4.21	4.65	4.62	5.65	5.66	3.38	3.98	4.38	2.68	4.94	4.07	3.81	2.53

Table DR4.

Organic carbon isotope of the analyzed mudstones from Xiahuayuan and Beipiao successions. (Height: m)

Sample	XHY01	XHY02	XHY03	XHY04	XHY05	XHY06	XHY07	XHY08	XHY09	XHY10	XHY11	XHY12	XHY13	XHY14	XHY15	XHY16	XHY17	XHY18	XHY19	XHY20	XHY21	XHY22
Height	142.1	140.03	138.15	136.32	134.26	132.77	126.05	124.1	122.03	120.19	116.22	112.11	110.06	108.02	105.89	102.05	100.01	94.9	92.09	89.1	86.22	82.03
$\delta^{13}C_{org}$	-23.912	-23.875	-24.215	-24.532	-24.985	-25.032	-23.984	-24.578	-24.457	-24.742	-24.511	-24.693	-24.291	-24.739	-25.146	-23.951	-23.846	-25.695	-25.984	-23.782	-23.915	-23.124
Amt%	0.817	0.987	1.012	1.003	1.006	0.077	0.068	0.068	0.096	0.061	0.062	0.064	0.06	0.066	0.077	0.056	0.058	0.057	0.052	0.371	0.65	1.376

Sample	XHY23	XHY24	XHY25	XHY26	XHY27	XHY28	XHY29	XHY30	XHY31	XHY32	XHY33	XHY34	XHY35	XHY36	XHY37	XHY38	XHY39	XHY40	XHY41	XHY42	XHY43	XHY44
Height	78.14	75.12	72.02	67.99	64.88	61.02	57.88	53.98	52.09	50.11	48.19	46.02	44.12	42.11	40.02	39.11	38.06	36.12	34.03	32.08	30.11	28.1
$\delta^{13}C_{org}$	-23.535	-22.892	-23.323	-23.148	-23.635	-23.604	-23.89	-23.782	-23.499	-23.745	-23.759	-23.871	-23.563	-23.693	-23.649	-23.561	-23.654	-23.959	-23.62	-23.636	-23.417	-23.596
Amt%	0.83	3.028	2.14	1.205	0.973	0.881	1.534	1.023	1.002	1.016	1.86	2.375	1.756	1.214	0.817	2.046	2.065	1.466	1.478	1.246	1.431	1.84

Sample	XHY45	XHY46	XHY47	XHY48	XHY49	XHY50	XHY51	XHY52	XHY53	XHY54	XHY55	XHY56	XHY57	BP01	BP02	BP03	BP04	BP05	BP06	BP07	BP08
Height	26.23	23.88	20.12	18.01	15.77	13.98	12.13	10.11	8.21	5.88	4.02	1.98	0	96.04	94.24	91.98	90.22	88.17	86.12	84.22	82.13
$\delta^{13}C_{org}$	-23.591	-22.552	-23.138	-23.086	-23.409	-23.499	-23.326	-23.288	-22.769	-23.569	-24.056	-23.86	-24.154	-25.21	-24.96	-25.346	-25.384	-25.481	-24.646	-25.464	-25.435
Amt%	2.518	1.965	2.435	1.976	1.336	1.415	1.567	3.091	1.015	2.843	2.407	0.616	0.056	0.039	0.103	0.087	0.045	0.102	0.087	0.093	0.07

Sample	BP09	BP10	BP11	BP12	BP13	BP14	BP15	BP16	BP17	BP18	BP19	BP20	BP21	BP22	BP23	BP24	BP25	BP26	BP27
Height	80.23	78.11	76.04	72.01	69.93	66.21	64.17	62.32	59.11	58.04	56.14	55.16	54.13	53.21	52.23	51.12	50.22	48.04	43.97
$\delta^{13}C_{org}$	-25.678	-26.483	-26.428	-25.523	-25.732	-24.182	-25.352	-25.438	-24.602	-25.572	-24.622	-25.14	-25.004	-25.714	-24.454	-24.959	-24.349	-25.359	-25.526
Amt%	0.068	0.071	0.044	0.083	0.044	0.156	0.03	0.058	0.101	0.045	0.065	0.074	0.059	0.106	0.152	0.049	0.056	0.089	0.109

Sample	BP28	BP29	BP30	BP31	BP32	BP33	BP34	BP35	BP36	BP37	BP38	BP39	BP40	BP41	BP42	BP43	BP44	BP45
Height	40.03	35.88	34.13	32.11	28.12	24.05	22.13	21.21	20.23	19.21	18.22	14.1	10.05	8.13	6.22	5.13	4.11	0
$\delta^{13}C_{org}$	-25.679	-25.66	-25.855	-26.228	-26.108	-26.017	-25.472	-25.214	-25.953	-25.116	-22.864	-24.25	-24.916	-22.92	-25.679	-25.682	-25.353	-24.586
Amt%	0.051	0.034	0.093	0.094	0.108	0.07	0.068	0.071	0.092	0.073	0.042	0.059	0.074	0.062	0.103	0.061	0.059	0.649
